# Chemostratigraphic correlations across the first major trilobite extinction and faunal turnovers between Laurentia and South China

**DOI:** 10.1038/s41598-019-53685-2

**Published:** 2019-11-22

**Authors:** Jih-Pai Lin, Frederick A. Sundberg, Ganqing Jiang, Isabel P. Montañez, Thomas Wotte

**Affiliations:** 1 Department of Geosciences, National Taiwan University, P.O. Box 097, Taipei, 10699 Taiwan; 2 Museum of Northern Arizona, 3101 N. Fort Valley Road, Flagstaff, AZ 86001 USA; 3 Department of Geoscience, University of Nevada, Las Vegas, NV 89154-4010 USA; 4 Department of Earth and Planetary Sciences, University of California, Davis, One Shields Avenue, Davis, CA 95616 USA; 5 Institut für Geologie, Technische Universität Bergakademie Freiberg, Bernhard-von-Cotta-Straße 2, D-09599 Freiberg, Germany

**Keywords:** Geochemistry, Stratigraphy

## Abstract

During Cambrian Stage 4 (~514 Ma) the oceans were widely populated with endemic trilobites and three major faunas can be distinguished: olenellids, redlichiids, and paradoxidids. The lower–middle Cambrian boundary in Laurentia was based on the first major trilobite extinction event that is known as the Olenellid Biomere boundary. However, international correlation across this boundary (the Cambrian Series 2–Series 3 boundary) has been a challenge since the formal proposal of a four-series subdivision of the Cambrian System in 2005. Recently, the base of the international Cambrian Series 3 and of Stage 5 has been named as the base of the Miaolingian Series and Wuliuan Stage. This study provides detailed chemostratigraphy coupled with biostratigraphy and sequence stratigraphy across this critical boundary interval based on eight sections in North America and South China. Our results show robust isotopic evidence associated with major faunal turnovers across the Cambrian Series 2–Series 3 boundary in both Laurentia and South China. While the olenellid extinction event in Laurentia and the gradual extinction of redlichiids in South China are linked by an abrupt negative carbonate carbon excursion, the first appearance datum of *Oryctocephalus indicus* is currently the best horizon to achieve correlation between the two regions.

## Introduction

The international correlation of the traditional lower–middle Cambrian boundary has been exceedingly difficult primarily due to apparent diachroniety of the datum species used to define the boundary reflecting the endemic faunas. Traditionally, this boundary has been marked by the first appearance datum (FAD) of *Paradoxides* (and other paradoxidid trilobites) in western Gondwana, Baltica, and Siberia^1–5^, the last appearance datum (LAD) of *Redlichia* (and other redlichiid trilobites) in South China^6,7^, and the LAD of *Olenellus* (and other olenellid trilobites) in Laurentia^8,9^. However, it has become apparent that the FAD of *Paradoxides* is not synchronous in Gondwana^10–14^, and this boundary is earlier than either the LADs of *Redlichia* or *Olenellus*^15^. In addition, the presumed synchronicity of the LADs of *Redlichia* or *Olenellus* also appears to be questionable^15–17^. The three major trilobite faunas discussed here are: olenellids, redlichiids, and paradoxidids. Olenellids include taxa in the families Olenellidae and Biceratopsidae and are confined to the paleo-continent Laurentia. Redlichiids used herein include members in the subfamily Redlichiinae, and they are found in eastern Gondwana, North China, and South China. These two trilobite stocks are separated from each other by the paradoxidids, including all species within the genus *Paradoxides*, which occur in West Gondwana, Avalonia, Siberia, and Baltica.

In an attempt to provide an accurate correlation near the traditional lower–middle Cambrian boundary, the IUGS has recently approved the Global Boundary Stratotype Section and Point (GSSP) for the Miaolingian Series and Wuliuan Stage, with their bases replacing the traditional boundary^18^. The base of the Wuliuan Stage is based on the FAD of *Oryctocephalus indicus*^19^ in the Kaili Formation, Wuliu-Zengjiayan section, Jianhe County, Guizhou Province, China^18,20–24^. This base coincides with the base of Miaolingian Series. Sundberg *et al*.^25^ documented the abrupt faunal turnover at the GSSP section. While this datum is useful for correlating strata in South China, India, North Korea, Siberia, and Laurentia, it does not occur in western Gondwana, Baltica, and Avalonia^15,26^.

**Figure 1:**
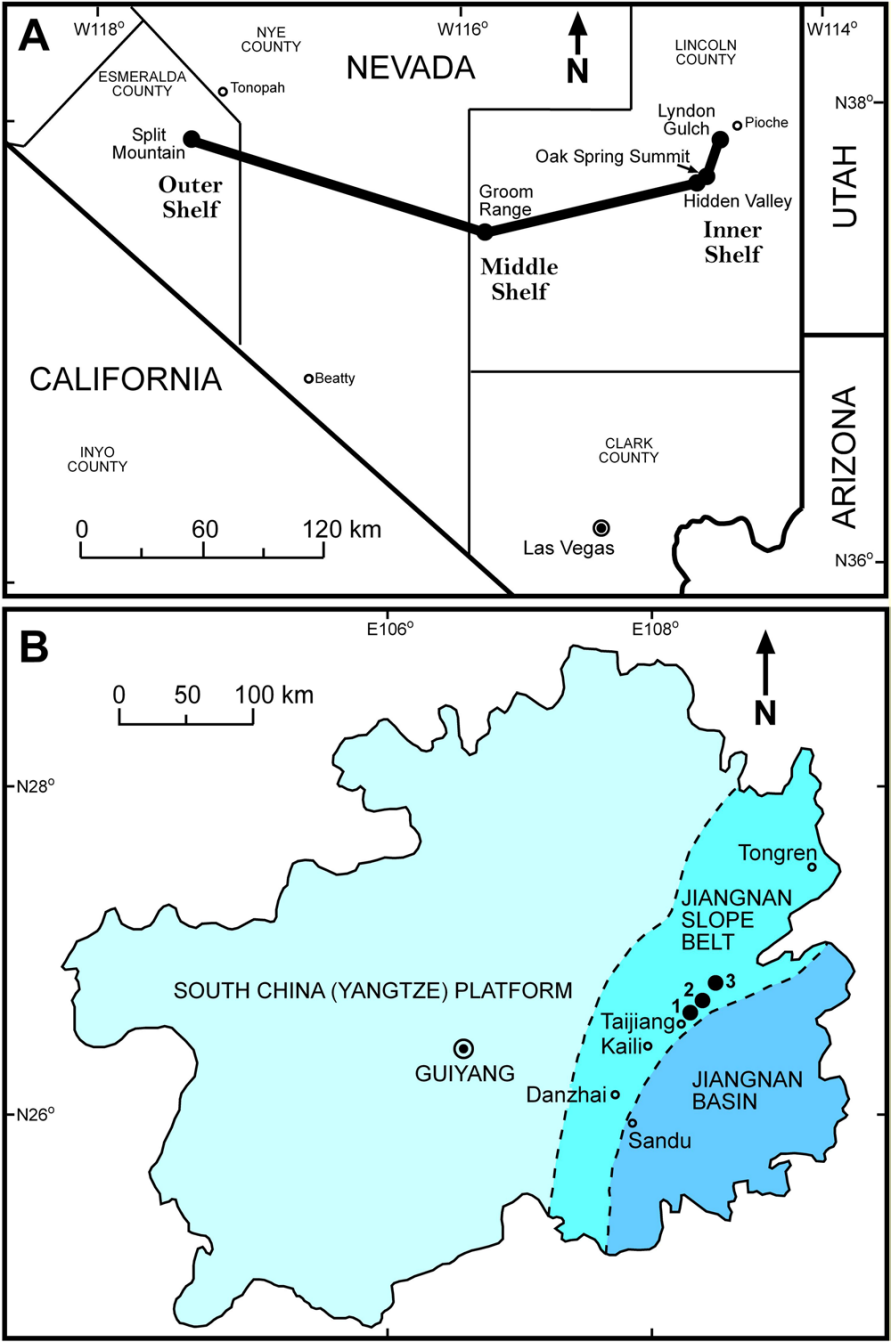
Location map of sections sampled for carbon isotopes in (**A**) Nevada and the fence panel linking these sections in Fig. 3 and Supplementary Fig. 9 and (**B**) reported localities^47^ of the Kaili Formation and Kaili-equivalent units in South China. 1 – Wuliu-Zengjiayan, 2 – Miaobanpo, and 3 – Jianshan sections.

The goals of this paper are to provide an accurate correlation of the base of the Miaolingian Series and to evaluate whether the redlichiids and olenellids became extinct synchronously. A high-resolution biostratigraphically constrained carbon isotope chemostratigraphy compiled from eight stratigraphic successions spanning the extinctions of the olenellids, redlichiids and the FAD of *O. indicus* across the paleo-shelf of Nevada (Fig. 1A), USA and Guizhou, South China is presented here. Montañez *et al*.^27^ first reported a prominent negative carbon excursion at or close to the base of the traditional lower–middle Cambrian boundary in Laurentia, and this excursion has been designated as the Redlichiid-Olenellid Extinction Carbon isotope Excursion (ROECE)^28,29^. By comparing the western U.S. record to a compilation of new and previously reported data from Guizhou, South China (Fig. 1B), our goal is to construct a global carbon chemostratigraphy by integration of the chemo- and biostratigraphic data from the two regions. The new chemostratigraphic correlation presented here should permit correlation of this critical interval to other regions that lack *O. indicus* and associated faunas (e.g., Europe, Morocco, Australia). Our study represents the most comprehensive chemostratigraphy and biostratigraphy across the Cambrian Series 2–Miaolingian boundary interval from Nevada and South China for testing the synchronicity of the olenellid extinction in Laurentia and redlichiid extinction in Gondwana.

## Geologic Settings

**Figure 2:**
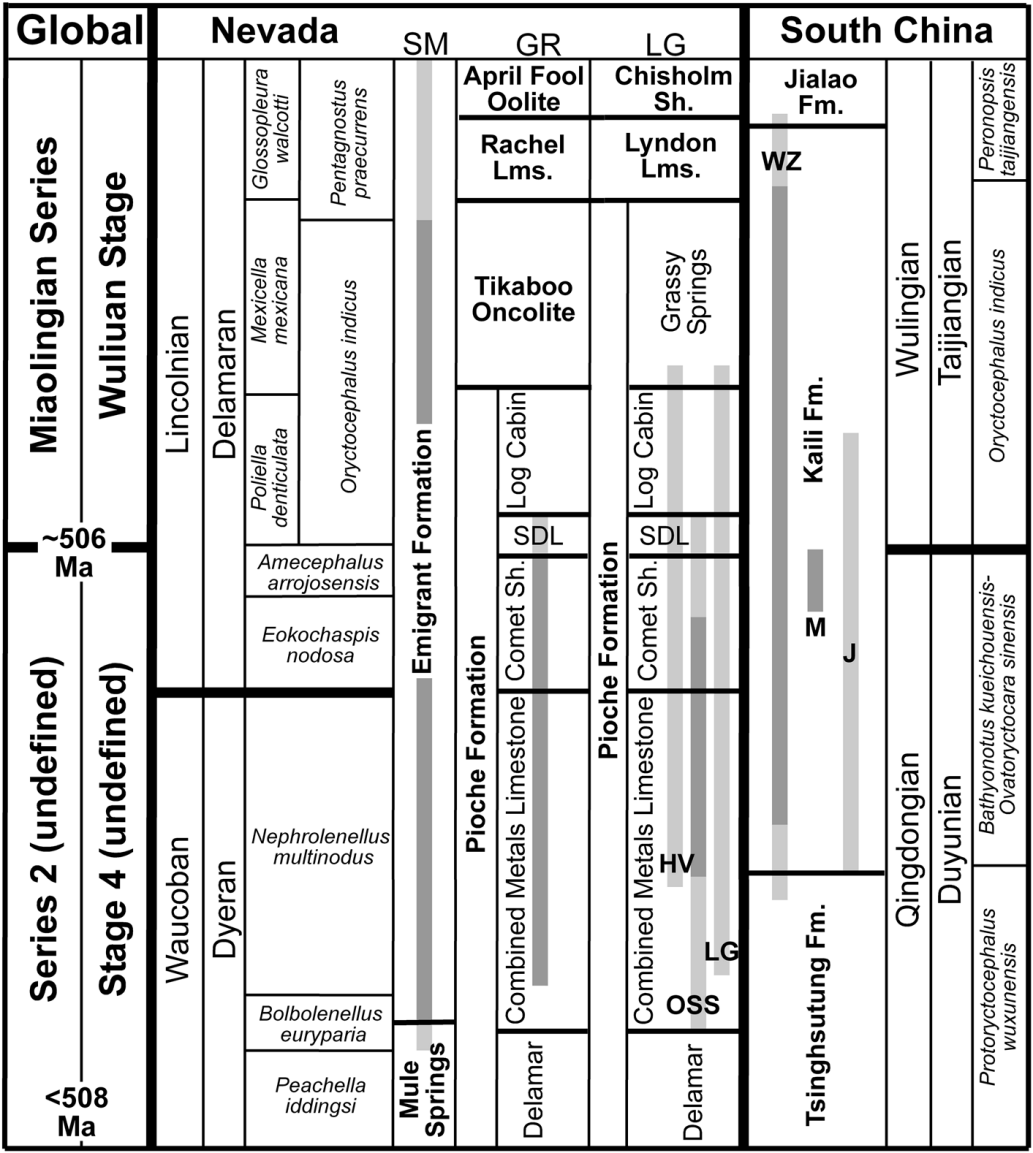
Correlation chart of sections sampled for δ^13^C_carb_, δ^18^O_carb_, and δ^13^C_org_ in Nevada and in South China. Light gray strip = δ^13^C_carb_ and δ^18^O_carb_ sample range; darker gray strip = both δ^13^C_carb_ and δ^13^C_org_ sample range. SM = Split Mountain section; GR = Groom Range section; LG = Lyndon Gulch section; HV = Hidden Valley section; OSS = Oak Spring Summit section; SDL = Susan Duster Limestone member; Mule Springs = Mule Springs Limestone; WZ = Wuliu-Zengjiayan section; M = Miaobanpo section; J = Jianshan section.

**Figure 3:**
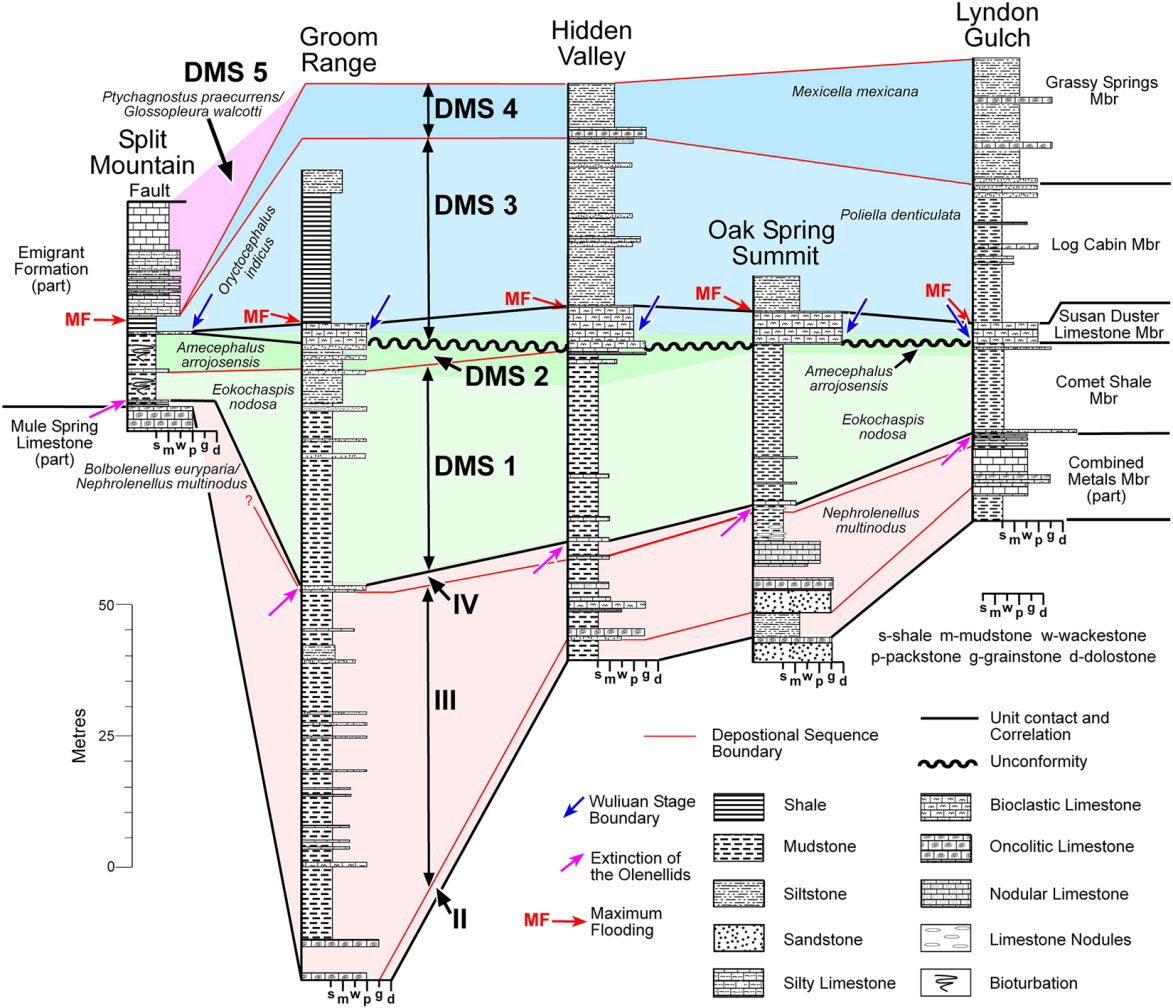
Stratigraphic sections in Nevada sampled for carbon isotopes with biostratigraphic (color bands), sequence (thin orange lines; data from refs^32,34^) and lithostratigraphic (thicker black lines) correlations. Sections are hung on the base of the *Oryctocephalus indicus* Biozone and its correlative base of the *Poliella denticulata* Biozone, which marks the base of the Wuliuan Stage and Miaolingian Series. DMS 1–5: Delamaran sequence 1 to 5 (ref.^32^); I–IV: Dyeran sequence I to IV (ref.^34^). Revised age estimate for the base of Wuliuan Stage in Laurentia is based on the recent work by Karlstrom et al. (ref.^92^).

The detailed lithostratigraphic and biostratigraphic correlations across the Cambrian shelf from previous studies (Figs 1, 2, 3)^16,17,21,30–35^ provide a context for defining carbon isotope excursions in the Miaolingian Series, Wuliuan Stage boundary interval in Nevada. Five sections distributed in eastern (Oak Spring Summit, Hidden Valley, and Lyndon Gulch) and central (Groom Range) Nevada as well as the uppermost Mule Spring and lower Emigrant formations of western Nevada (Split Mountain) were sampled for carbonate (δ^13^C_carb_) and organic carbon (δ^13^C_org_) isotope analysis, with targeted intervals being the Combined Metals, Comet Shale, Susan Duster Limestone, Log Cabin, and Grassy Springs members of the Pioche Formation (Fig. 2).

Webster^34,35^ and McCollum & McCollum^33^ have subdivided the uppermost Dyeran to Delamaran strata of the western Laurentian margins into nine sequences (Fig. 3). Webster^34^ has identified four sequences within the lower portion of the Pioche Formation. Sequence I is represented by the Delamar Member, Pioche Formation, in the middle and inner shelf region and the upper Harkless Formation and the Mule Spring Limestone in the outer shelf region. This sequence consists of the *Arcuolenellus arcuatus* Biozone to lowermost *Bolbolenellus euryparia* Biozone. Sequence II is represented by the lower portion of the Combined Metals Member and the upper part of the Mule Spring Limestone, and is within the *Bolbolenellus euryparia* Biozone. Sequence III is represented by the upper portion of the Combined Metals Member and occurs within the upper *Bolbolenellus euryparia* and *Nephrolenellus multinodus* biozones. Sequence IV is represented by the uppermost portion of the Combined Metals Member and is within the upper *Nephrolenellus multinodus* Biozone. This sequence is thin, approximately 0.5 to 1.0 m, occurring between the last “ribbon” limestone with olenellids and the first “ribbon” limestone containing faunas from the *Eokochaspis nodosa* Biozone (“boundary limestone”), and contains the last olenellid trilobites in the region. Sequences III and IV cannot be differentiated in the Split Mountain section.

McCollum & McCollum^33^ separate the sequences based on abrupt facies changes, unconformities, and transgressive packages (Fig. 3). Sequence DMS 1 begins at the base of the Comet Shale Member and the *Eokochaspis nodosa* Biozone. McCollum & McCollum^33^ have suggested this sequence lies on top of a disconformity based on different lithologies of the Combined Metals Member under the “boundary limestone” that is at the base of DSM 1. Webster^34^ provides an alternative interpretation, suggesting that the persistence of sequence IV underneath the “boundary limestone” argues against a large unconformity at the traditional Laurentian lower–middle Cambrian boundary. Trilobite occurrences^36,37^ also indicate a conformable boundary. Sequence DMS 2 begins at the base of the limestone, 9 m above the base of the Emigrant Formation and at the beginning of the *Amecephalus arrojosensis* Biozone at the Split Mountain section (Fig. 3). This sequence has been greatly reduced in the middle and inner shelf due to the disconformity at the base of the Susan Duster Limestone. Sequence DMS 3 begins at the base of the Susan Duster Limestone and the upper *A. arrojosensis* Biozone. This sequence consists of the Susan Duster Limestone and Log Cabin members and contains the uppermost *A. arrojosensis*, *O. indicus*, and *P. denticulata* biozones. DMS 4 begins at the base of the Grassy Springs Member, Pioche Formation and the *M. mexicana* Biozone. This sequence was only sampled for isotope analyses in the Hidden Valley and Lyndon Gulch sections. DMS 3 and 4 cannot be separated in the Split Mountain section. DMS 5 was only sampled in the Split Mountain section covering the limestones containing the *P. praecurrens/G. walcotti* biozones.

The biostratigraphic framework used in this study (Figs 2 and 3) is derived from the detailed work of Webster^34^, McCollum & Sundberg^38^, and Sundberg^21,39^. Webster^34^ recognized six olenellid biozones for the upper Dyeran Stage (traditional upper lower Cambrian), of which two are present in this study: the upper *B. euryparia* and *N. multinodus* biozones. Sundberg^21,39^ recognized six biozones for the Delamaran Stage (traditional lower–middle Cambrian), of which the *E. nodosa*, *A. arrojosensis*, *P. denticulata*, and *M. mexicana* biozones are present in the middle to inner shelf sections. In the outer shelf, the *O. indicus* Biozone overlies the *A. arrojosensi*s Biozone and is equivalent in age to the *P. denticulata* and probably most of the *M. mexicana* biozones. Overlying the *O. indicus* Biozone are the *P. praecurrens/G. walcotti* biozones^39^. These biozones provide a more refined framework than that presented by Palmer & Halley^40^ that was used by previous isotopic studies^27,41,42^. Based on trilobite species and genera, the base of the Miaolingian Series and Wuliuan Stage is the base of the *O. indicus* Biozone and its correlative *P. denticulata* Biozone.

The studied stratigraphic sections in eastern Guizhou Province, South China contain the upper Tsinghsutung (also known as Qingxudong), Kaili, and lower Jialao formations (Figs 1B and 2). Three sections were sampled, in a southwest to northeast direction, the Wuliu-Zengjiayan^43,44^, the Miaobanpo^45^, and the Jianshan^44^ sections. The Wuliu-Zengjiayan section of the Kaili Formation has been selected as the GSSP of the Miaolingian Series and Wuliuan Stage^18^. The Kaili Formation is dominated by shales with abundant micritic beds in its upper portion^46^. Similar micritic beds in the lower portion of the formation contain possibly post-depositional carbonate cements and concretions formed in mudstones. The Kaili Formation was deposited below storm wave base on the Jiangnan Slope^47^ with features indicative of gravity sliding^25^, where suspended mud settled down from the water column^46^. The underlying Tsinghsutung and overlying Jialao formations are predominantly dolostones.

The biostratigraphic scheme for the three formations (Fig. 2) is based on (in ascending order): *Protoryctocephalus wuxunensis*, *Bathynotus kueichouensis*-*Ovatoryctocara sinensis*, *Oryctocephalus indicus*, and *Peronopsis taijiangensis* biozones^20,22–24,48,49^. The latter two zones are defined by the FAD of the named taxa. Trilobites have not been reported from the upper dolostones of the Tsinghsutung Formation, however, they have been assigned to the *P. wuxunensis* Biozone^49,50^. Trilobites from the middle portion of the Jialao Formation consist of *Solenopleuropsis*, *Jialaopsis*, *Kootenia*, and *Parafuchouia* (19 m to 72 m above the base^51^); these taxa overlap with the *Sunaspis*-*Sunaspidella* Biozone of the middle Hsuchuangian Regional Stage^52^.

## Results

### Trilobite faunas

**Figure 4:**
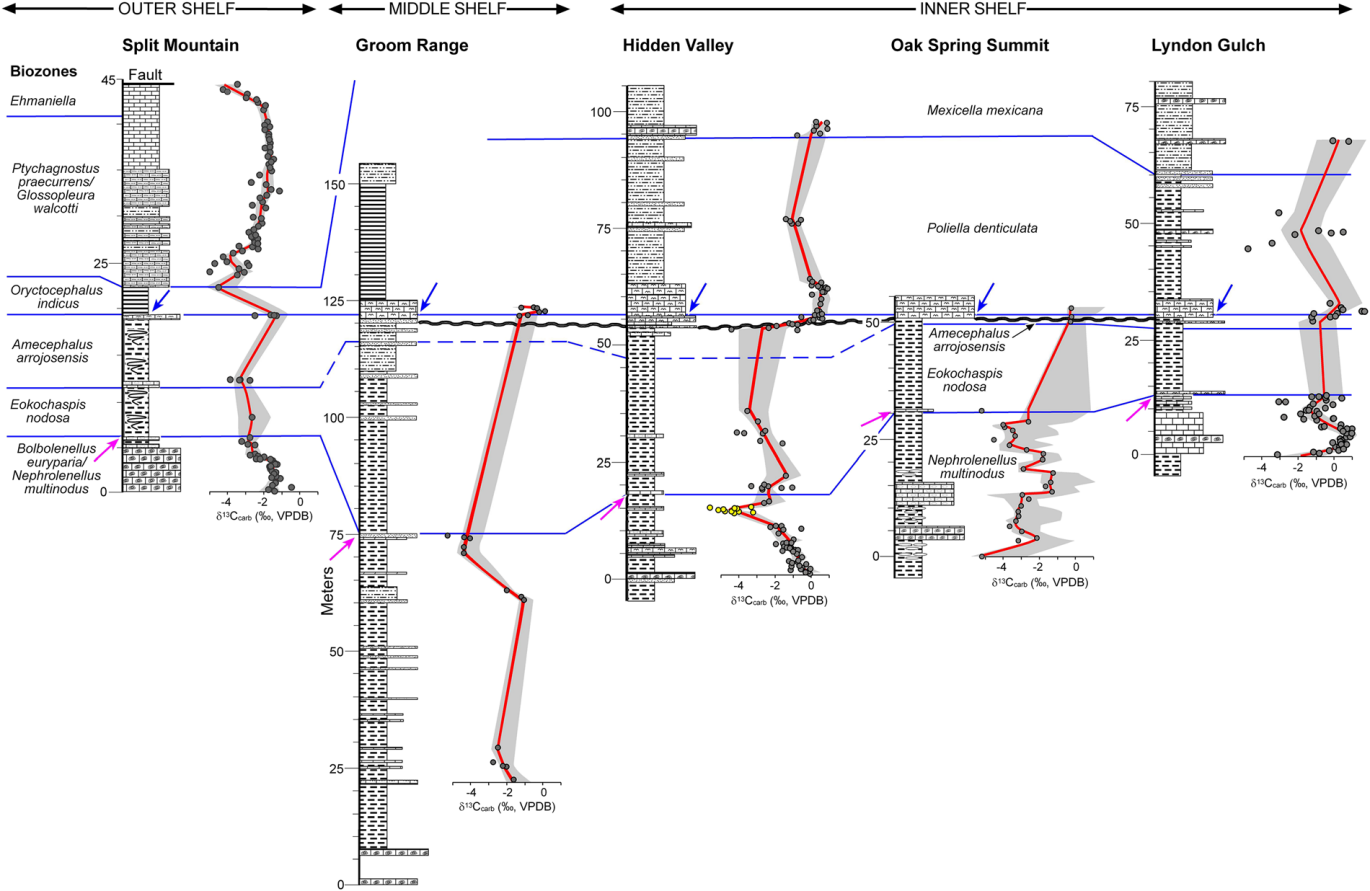
The δ^13^C_carb_ results from the five sections in Nevada (Oak Spring Summit data from ref.^42^ and uses their measured section). Red line is the LOESS curve for each section. Sections are hung on the base of the *Oryctocephalus indicus* Biozone and its correlative base of the *Poliella denticulata* Biozone, which marks the base of the Miaolingian Series, Wuliuan Stage. The Split Mountain section is presented at twice the vertical scale of the other sections.

**Figure 5:**
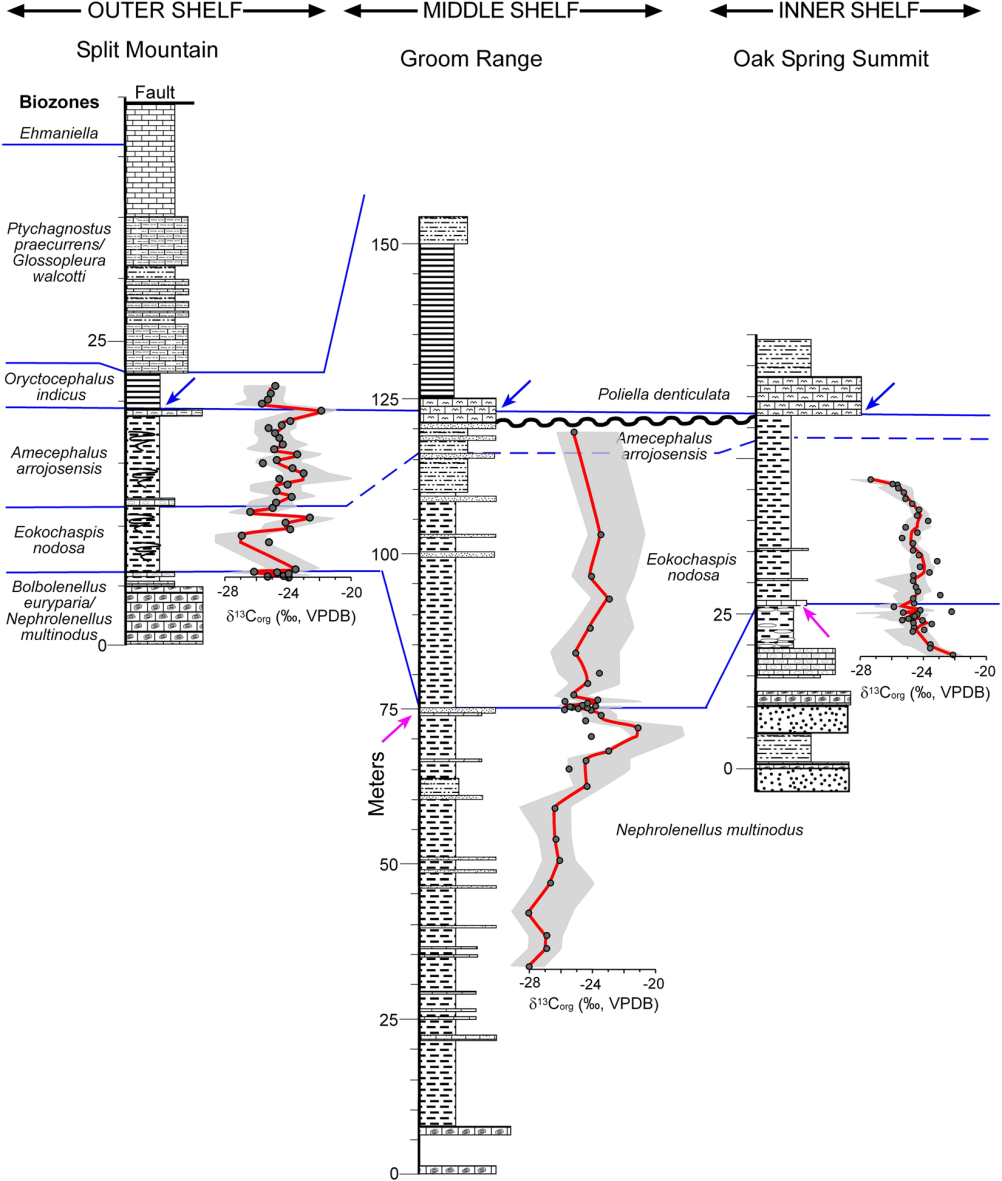
The δ^13^C_org_ results from the Split Mountain, Groom Range, and Oak Spring Summit sections in Nevada. Red line is the LOESS curve generated using 0.1 curvature. Sections are hung on the base of the *Oryctocephalus indicus* Biozone and its correlative base of the *Poliella denticulata* Biozone, which marks the base of the Miaolingian Series, Wuliuan Stage. The measured section for Oak Spring Summit is based on measurements from Webster^35^ and L.B. & M. McCollum (personal communications).The Split Mountain section is presented at twice the vertical scale of the other sections.

Trilobite evolutionary faunas, known as biomeres, define most of the Laurentian Cambrian series and stage boundaries^53^. Based on the current concepts for the nomenclature for Cambrian stages of Laurentia, the Dyeran Stage coincides with the Olenellid Biomere and the Delamaran Stage is referred as the interval of the Corynexochid Biomere. The Dyeran–Delamaran stage boundary coincides with the Olenellid-Corynexochid faunal turnover and carbon isotope anomalies. The Laurentian olenellid extinction event is difficult to correlate with other paleo-continents due to a lack of olenellids in other cratons (Supplementary Fig. S11B–D). Alternatively, the extinction of redlichiid trilobites (Supplementary Fig. S11V,W) in South China has been interpreted as synchronous with the olenellid extinction event in Laurentia^54^. Recently, the GSSP for both the Stage 5 (now Wuliuan Stage) and Series 3 (Miaolingian Series) has been defined and is based on the FAD of *Oryctocephalus indicus* (Fig. 2 and Supplementary Fig. S11E–G)^18^. This datum is immediately above the extinction event of redlichiid trilobites in South China. In Laurentia, however, as exemplified by the Nevada sections studied here, there are two biostratigraphic units: the *E. nodosa* Biozone and the *A. arrojosensis* Biozone above the olenellid extinction and below the FAD of *O. indicus* (Figs 4 and 5). Those two biozones seem to be regional. In particular, the *A. arrojosensis* Biozone is often truncated among strata in eastern Nevada due to the occurrence of a disconformity (Fig. 3), but this biozone is also present in California, Mexico, and Argentina^30,55,56^.

### Carbonate carbon and oxygen isotopes

Figure 4 and Supplementary Figs S2–S6 illustrate the distribution of δ^13^C_carb_ values for the five sections sampled across the Cambrian Nevadan shelf in this study. The data are mostly limited to carbonate beds, which are unevenly distributed in the shale-dominated intervals. Biostratigraphic (blue lines in Fig. 4 and Supplementary Figs S2–S6) and lithostratigraphic correlations (Fig. 3) provide the control for the regional correlation of δ^13^C_carb_ excursions. In the Split Mountain section (Supplementary Fig. S2), for instance, δ^13^C_carb_ values of the upper Mule Spring Limestone to lowermost Emigrant Formation decrease upward from approximately −1.0‰ to −2.5‰ and maintain around −2.7‰ through the base of the *A. arrojosensis* Biozone. An increase in δ^13^C_carb_ occurs at the top of the *A. arrojosensis* Biozone with values of approximately −1.0‰. From the *P. praecurrens*/*G. walcotti* Biozone to the lower *Ehmaniella* Biozone, δ^13^C_carb_ values begin around −4.1‰, increase to −1.1‰ half way through the strata, and subsequently decrease to −3.8‰.

In South China, the new δ^13^C_carb_ data for the Wuliu-Zengjiayan section include samples in the upper and lower portions of the Kaili and adjacent formations (Supplementary Fig. S8). This new dataset is in general agreement with that provided by Guo *et al*.^44^ with discrepancies seen in the upper carbonate layers of the Kaili Formation. Guo *et al*.^44^ had few data points in this portion of the section and the δ^13^C_carb_ values are generally between +1.0‰ and +2.0‰. The new data for this portion of the section range from −1.0‰ to +1.0‰. In the Wuliu-Zengjiayan section, δ^13^C_carb_ values of the uppermost Tsinghsutung Formation decrease from +2.1‰ to −0.7‰. This decreasing trend of δ^13^C_carb_ values continues stratigraphically upward until the base of the Kaili Formation (−1.9‰), and then it shifts toward positive and increases abruptly to +3.1‰ in the lowermost 5 m of the Kaili Formation. From this level, δ^13^C_carb_ values decrease to −3.6‰ around the *B. kueichouensis-O. sinensis*/*O. indicus* boundary and vary between −2.4‰ and 2.0‰ (mean = −0.3‰) for the remaining portion of the Kaili Formation. The Wuliu-Zengjiayan section has δ^18^O_carb_ values from −9.9‰ to −3.6‰. A decreasing trend from −6.1‰ to −9.9‰ is present from the uppermost Tsinghsutung Formation to the *B. kueichouensis-O. sinensis*/*O. indicus* boundary. After some fluctuations between −9.9‰ and −7.1‰ around the boundary, δ^18^O_carb_ values are mostly in the range of −8‰ to −6‰, with a few higher values between −5.8‰ and −3.6‰. Overall, there is no correlation between δ^13^C_carb_ and δ^18^O_carb_ (Supplementary Fig. S1F).

In the Miaobanpo section (Supplementary Fig. S9A), δ^13^C_carb_ values from a 5-m-thick interval below the FAD of *O. indicus* initially increase from −3.1‰ to −0.5‰ and then decrease to minimum values of −7.8‰ to −6.1‰. At the same interval, δ^18^O_carb_ values increase from −8.6‰ to −6.2‰. In the Jianshan section (Supplementary Fig. S9B) (replotted from Guo *et al*.^44^), δ^13^C_carb_ values show a positive shift with highest values up to +3.1‰ in the lowermost 10 m of the Kaili Formation, followed by a negative shift with minimum values down to −6.9‰ across the *B. kueichouensis-O. sinensis*/*O. indicus* boundary. The δ^18^O_carb_ values vary from −9.9‰ to −2.5‰, showing a positive shift corresponding to the negative δ^13^C_carb_ shift across the *B. kueichouensis-O. sinensis*/*O. indicus* boundary. There is no general δ^13^C_carb_ –δ^18^O_carb_ co-variation in the Miaobanpo and Jianshan sections (Supplementary Fig. S1G,H).

### Organic carbon isotopes

In the Split Mountain section, organic carbon isotope values vary between −27.1‰ and −23.7‰ throughout the lower portion of the section, showing frequent 3–4‰ shifts at the interval from the *E. nodosa* Biozone to *O. indicus* Biozone (Supplementary Fig. S2). There is no apparent correlation between δ^13^C_carb_ and δ^13^C_org_ for the sampled interval, except for the positive shift near the boundary of *A. arrojosensis* and *O. indicus* biozones. Given the general lack of carbonates in this portion of the section there may be more δ^13^C_carb_ variations that were not documented.

In the Groom Range section (Supplementary Fig. S3), organic carbon isotope values vary between −28.3‰ and −21.4‰ throughout the lower half of the section. The Combined Metals Member illustrates an increase in δ^13^C_org_ from −28.3‰ to −23.3‰ approximately five meters below the contact between the Combined Metals Member and Comet Shale Member and the *N. multinodus*/*E. nodosa* biozone boundary. Above this interval, the δ^13^C_org_ values decrease towards −26.0‰ at the *N. multinodus*/*E. nodosa* boundary and then vary between −23.3‰ and −25.5‰ to the base of the *A. arrojosensis* Biozone. The δ^13^C_carb_ and δ^13^C_org_ seem to show opposite trends, but again the lack of paired δ^13^C_carb_ and δ^13^C_org_ data prevents a precise, one-to-one correlation of the δ^13^C_carb_ and δ^13^C_org_ shifts.

**Figure 6:**
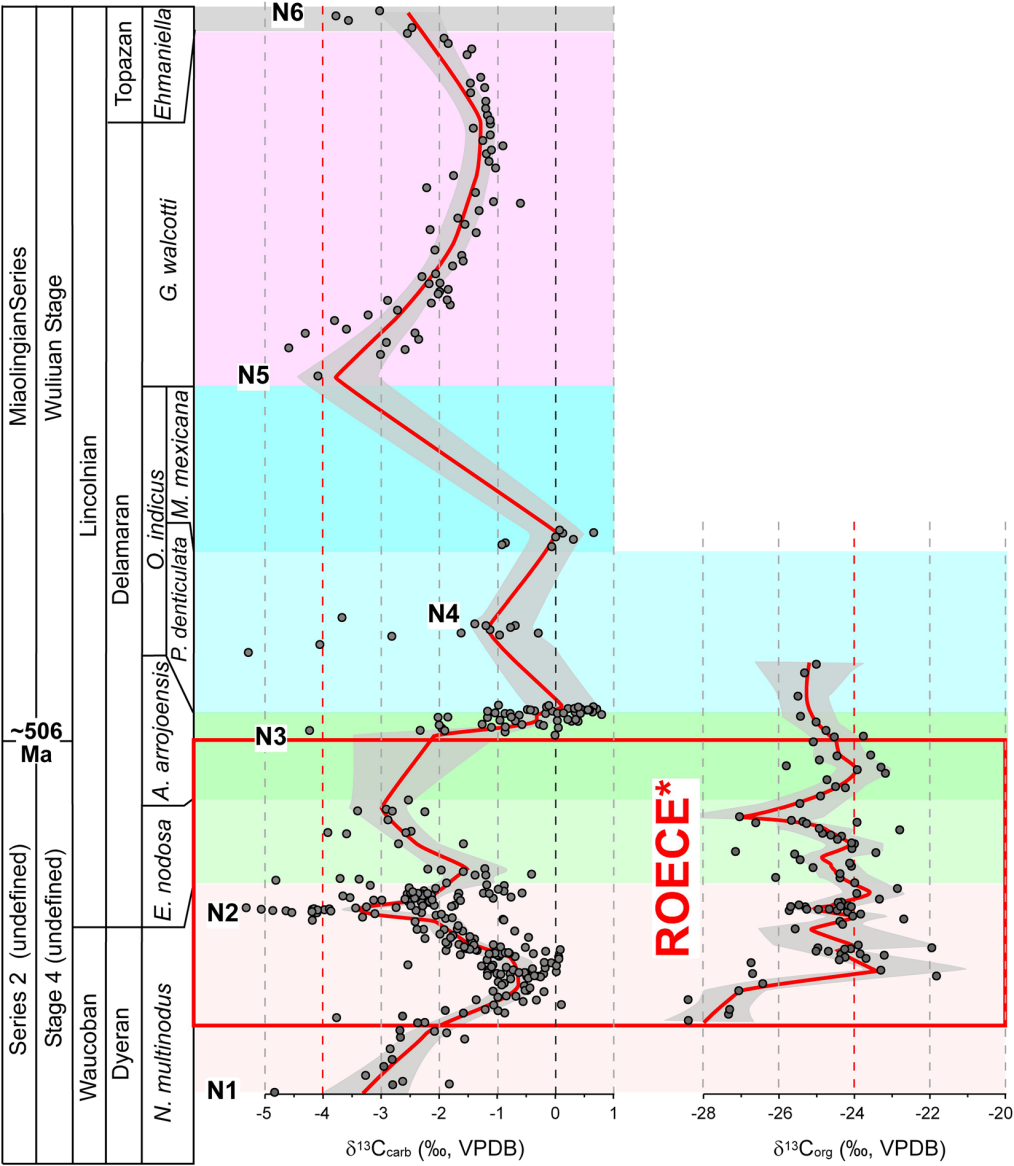
The δ^13^C_carb_ and δ^13^C_org_ summary curves based on the Nevada sections. ROECE* and red outlined area represents the expanded version of the Redlichiid-Olenellid Extinction Carbon isotope Excursion^28,29^. Vertical scale is based on relative biozone thicknesses.

Figure 5 illustrates the distribution of δ^13^C_org_ data from the three sections sampled across the Cambrian Laurentian shelf in this study. Due to the lack of paired carbonate and organic carbon isotope data, it is difficult to correlate every positive and negative shift in δ^13^C_carb_ and δ^13^C_org_. However, the negative shift in δ^13^C_carb_ at the *N. multinodus*/*E. nodosa* boundary (N2 in Fig. 6) is accompanied with lower δ^13^C_org_ values in both Groom Range and Oak Spring Summit sections. Such a correlation in the Split Mountain section is less obvious due to the lack of δ^13^C_org_ data below this interval.

In the South China data represented by the Wuliu-Zengjiayan section, δ^13^C_org_ values show an increasing trend from −32.0‰ to −24.5‰ from the basal Kaili Formation to the *B. kueichouensis-O. sinensis*/*O. indicus* boundary. This contrasts with the decreasing trend of δ^13^C_carb_ at the same stratigraphic interval. The middle portion of the Kaili Formation lacks δ^13^C_org_ data, and the upper portion shows a weak decreasing trend from −26.1‰ to −27.1‰, which is also not present in δ^13^C_carb_. In the Miaobanpo section (Supplementary Fig. S9A), δ^13^C_org_ values increase from −29.5‰ to −27.1‰. The magnitude of δ^13^C_org_ change (<2.5‰) is much smaller than that of δ^13^C_carb_ (up to 10‰) and they show opposite temporal trends.

In summary, three sections from Laurentian δ^13^C_org_ profiles are provided here: Split Mountain, Groom Range, and Oak Spring Summit sections. Their values range between −28‰ to −20‰, and define a small negative δ^13^C_org_ shift across the olenellid extinction event (the boundary between *N. multinodus* and *E. nodosa* biozones) (Fig. 5). In South China, δ^13^C_org_ values from Wuliu-Zengjiayan and Miaobanpo sections range between −32‰ and −24‰. In both sections, there is a general trend toward less negative values (from −32‰ to −26‰ in Wuliu-Zengjiayan section; from −29.5‰ to −27‰ in Miaobanpo section) (Supplementary Figs S8 and S9A).

### Chemostratigraphy

Secular variations in carbon isotopic values occur in both the Laurentian and South China sections. Most δ^13^C_carb_ values from the Laurentian sections are negative (Supplementary Figs S1–S6). Thus, only robust negative excursions are considered as having chemostratigraphic significance. The first δ^13^C_carb_ excursion (N1) in the studied sections is a negative shift (−5‰ to −3.5‰) in Laurentia (Fig. 6). The second robust negative excursion (N2) (down to −5‰) occurs near the LAD of olenellid trilobites and this excursion is present in all five sections (Figs 4, 6; Supplementary Figs S2–S6). The third excursion (N3) has minimum δ^13^C_carb_ values down to −4‰ and occurs within the *E. nodosa* Biozone (Fig. 6). The fourth excursion (N4) occurs in the middle *P. denticulata* Biozone. The fifth one (N5) occurs in the lower part of the *P. praecurrens* Biozone and the sixth δ^13^C_carb_ excursion (N6) occurs in the *Ehmaniella* Biozone (Fig. 6).

In the *N. multinodus* Biozone, there are two δ^13^C_carb_ shifts that can be identified (N1 and N2 (Fig. 6). The adjustment of the shifts is based on adding or subtracting the number of units that best align the two shifts from one section to another (e.g., Hidden Valley data was assigned to units 10–23 due to an incomplete sampling of the biozone). Alignment of data between N3 and N4 in the lower *P. denticulata* Biozone of the Susan Duster Limestone was also performed to determine the unit depth of data for the portion of *P. denticulata* and *A. arrojosensis* zones below and above this excursion. This was done by either adding or subtracting units from the matched excursion and readjusting the number of units back to the originally assigned number of units in the biozones.

**Figure 7:**
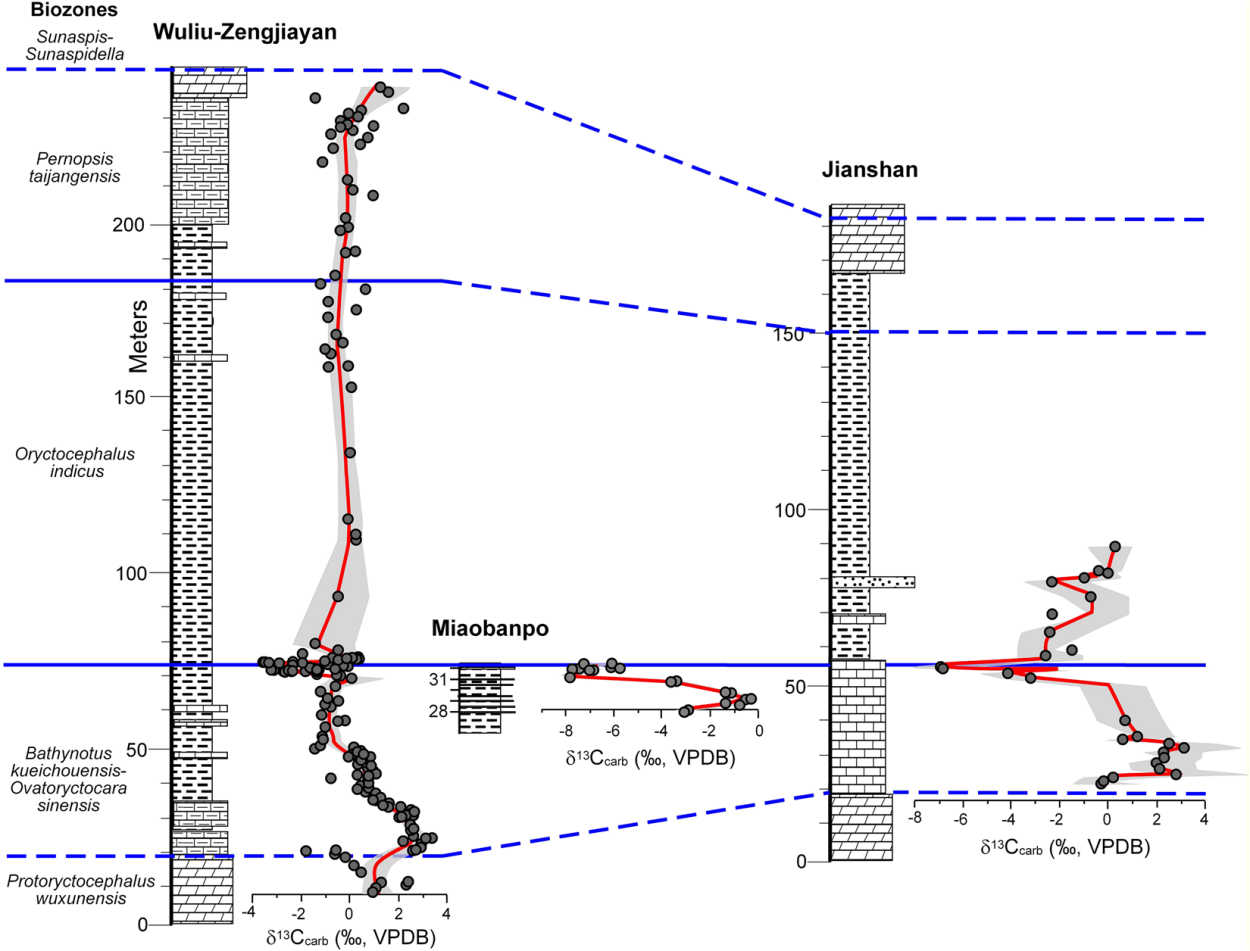
Stratigraphic sections in South China sampled for δ^13^C_carb_ with biostratigraphic (blue lines) correlations. Red line is the LOESS curve generated using 0.1 curvature. Sections are hung on the base of the *Oryctocephalus indicus* Biozone, which marks the base of the Miaolingian Series, Wuliuan Stage.

The lack of some of these shifts in one or more sections is primarily due to the lack of data from a portion of a section; for example, N1 is not apparent at the Split Mountain, Groom Range, and Hidden Valley sections probably due to the incomplete sampling of the basal parts of the Combined Metals Member and the Mule Spring Limestone. The absence of suitable carbonate to sample may also explain the absence of shifts or the full development of shifts in some sections. For example, N2 is well presented in the Lyndon Gulch, Hidden Valley, and Oak Spring Summit sections as a complete, 3–4‰ negative δ^13^C_carb_ excursion, but in the Split Mountain and Groom Range sections negative δ^13^C_carb_ values do not return back to higher values at the basal *Eokochaspis nodosa* Biozone.

In sections from South China, in contrast, only two negative shifts, N1 (−2.0‰) and N2 (close to −8.0‰), have been identified from the uppermost Tsinghsutung and lower Kaili formations (Fig. 7). The upper Kaili and lower Jialao formations do not show any significant change in δ^13^C_carb_, with most values between −1.0‰ and +1.0‰. Figure 7 illustrates δ^13^C_carb_ profiles for the Wuliu-Zengjiayan, Miaobanpo, and Jianshan sections. High resolution sampling of δ^13^C_carb_ across the traditional lower–middle Cambrian boundary interval among the three sections mentioned above provide additional constrains for intra-cratonic correlation (Supplementary Fig. S10). In the Miaobanpo section, a strong negative δ^13^C_carb_ excursion of −7.8‰ is present ~1.5 m below the FAD of *O. indicus*. This is consistent with the negative δ^13^C_carb_ excursion of −6.9‰ from the equivalent interval in the Jianshan section^44,45^. However, the δ^13^C_carb_ change from the GSSP section (Wuliu-Zengjiayan section) is only half of the magnitude, with the lowest value of −3.4‰ below the FAD of *O. indicus* (Supplementary Fig. S10). In summary, the second negative carbon excursion (N2) is robust and exhibited in all three studied sections (Fig. 7;  Supplementary Figs S8–S10) and is in close proximity with the extinction of redlichiid trilobites. Although strata from the Miaobanpo section (Supplementary Fig. S9A) were only sampled over five meters due to the lack of suitable carbonate-rich layers exposed in this section, it records a robust negative carbon excursion (down to −7.8‰) and represents the critical interval in between the FAD of *O. indicus* and the extinction of redlichiid trilobites^45^.

**Figure 8:**
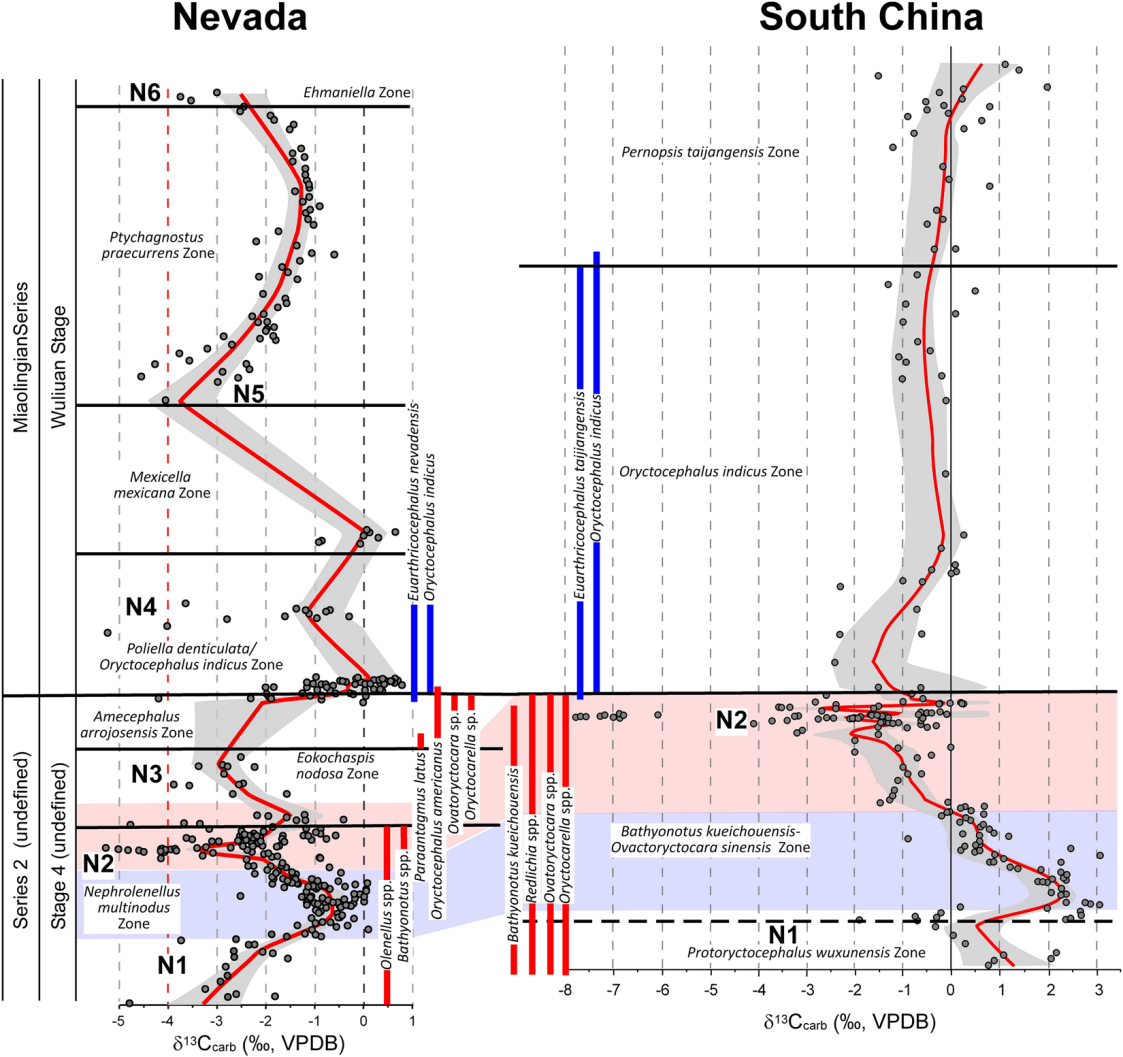
Correlation of summary δ^13^C_carb_ curves for Nevada and South China and their potential correlation based on the chemostratigraphy and trilobite biostratigraphy. Included are the stratigraphic ranges of key trilobite taxa occurring in the Miaolingian Series. Images of key trilobites are included in Supplementary Fig. S11.

## Discussion and Conclusions

### Diagenetic effect

Many researchers^57–61^ have addressed the potential effects of  post-depositional fluid-rock interaction in Precambrian and Paleozoic strata, and some data reported from Cambrian section indeed show strong meteoric diagenesis^62^. Bishop *et al*.^63^ studied rock samples from the Permian Capitan backreef and found that samples underwent meteoric diagenesis show the inverted J curve in an δ^18^O_carb_–δ^13^C_carb_ plot. For our samples there are no obvious linear trends among the data (Supplementary Fig. S1A–H). While the δ^18^O_carb_ values may be altered due to increasing burial temperatures in Paleozoic and older sedimentary strata, δ^13^C_carb_ values are thought to be temperature independent^61,64,65^. The decoupled δ^18^O_carb_ and δ^13^C_carb_ values and more importantly, the regionally persistent δ^13^C_carb_ shifts within bio- and lithostratigraphically controlled intervals suggest at least partial preservation of the marine carbon isotope record of chemostratigraphic significance.

### Carbonate and organic carbon isotopes

Positive correlations between δ^13^C_carb_ and δ^13^C_org_ values have been interpreted as evidence for changes in carbon cycling in the oceans^64,66^ in the geological record. Coupling of carbonate carbon and organic carbon isotopes has been reported for the positive carbon isotope excursion in the upper Cambrian known as the SPICE near the base of Furongian Series^67^. The coupling of δ^13^C_carb_ and δ^13^C_org_ data, however, are not evident in our sections, which are stratigraphically lower than the SPICE. Maloof *et al*.^68^ show that positive correlations between organic and inorganic carbon are likely to be associated with stratigraphic intervals indicating positive δ^13^C_carb_ excursions, whereas, they are decoupled in strata showing negative δ^13^C_carb_ excursions. Our results show that organic carbon and inorganic carbon isotopes are decoupled, similar to those across negative δ^13^C_carb_ excursions reported from lower Cambrian sections^68^. The origin of decoupled δ^13^C_carb_ and δ^13^C_org_ remains unresolved; possible causes may include contribution of dissolved organic carbon in the ocean^69^, recycled organic matter^70^, and/or detrital organic carbon^71^.

### Correlation between Laurentia and South China

Montañez *et al*.^27^ stated that “Our secular C isotope curve defines a previously undocumented, rapid (~100 k.y.), large-magnitude shift (≥4‰) to negative δ^13^C values in the terminal Early Cambrian. This negative C isotope excursion begins just prior to the oldest known mass extinction of trilobites…”. The trilobites referred to are the olenellids and this extinction marks the traditional boundary of the Laurentian lower–middle Cambrian. Dilliard *et al*.^41^ also recognized the onset of a negative δ^13^C_carb_ shift of probably similar magnitude in the Mackenzie Mountains, Northwest Territories, Canada, but that was based on only one sample associated with a flooding event, and several samples stratigraphically below. Chemostratigraphic correlations between Nevada and South China are compiled and interpreted in this study (Fig. 8). The well-defined negative shift N2 at the upper *Nephrolenellus multinodus* to basal *Eokochaspis nodosa* biozones in Nevada appears to correlate with the negative shift N2 in the uppermost *Bathynotus kueichouensis*-*Ovatoryctocara sinensis* Biozone, stratigraphically just below the FAD of *O. indicus* (Fig. 8) in South China. This is further supported by the strong shift toward less negative values that occur in the lower *N. multinodus* Biozone in Nevada and the shift toward positive values in the lower *B. kueichouensis*-*O. sinensis* Biozone (Fig. 8) in South China. Zhu *et al*.^28^ named this carbon excursion as “ROECE” based on Cambrian sections from China and interpreted that it is also associated with the redlichiid trilobite extinction in South China. This study questions the definition of “ROECE” based on the following reason. There are at least two negative shifts (N2 and N3) in between the LAD of olenellids and FAD of *O*. *indicus* recorded in Nevadan sections (ROECE* in Fig. 6 and Supplementary Fig. S7), and both of them correlate to the negative δ^13^C_carb_ shift in South China. Thus, the “ROECE” might not represent a single carbon excursion event as previously hypothesized^28^.

Correlation of the aforementioned negative δ^13^C_carb_ excursions from the two regions is in conflict with the taxa ranges from these sections (Fig. 8) for two reasons. First, the FAD of *O. indicus* occurs much higher in the section than the major N2 excursion in Nevada. As a partial solution to this mismatch, Zhao *et al*.^18^ have suggested that *Oryctocephalus americanus*^16^ (Supplementary Fig. S11H–J) is synonymous to *O. indicus* (Supplementary Fig. S11E–G) (also see the discussion in the Electronic Supplementary Material), thus lowering the FAD of *O. indicus* in Laurentia to the base of the *A. arrojosensis* Biozone (dashed line, Fig. 8). This slight adjustment, however, does not imply synchronicity of the two major δ^13^C_carb_ excursions. Second, the trilobites from the *A. arrojosensis* and/or *E. nodosa* biozones in Nevada include *Paraantagmus latus*^72^ (Supplementary Fig. S11U) and representatives of *Oryctocarella* (Supplementary Fig. S11Q,R) and *Ovatoryctocara* (Supplementary Fig. S11M–P). American specimens of *Ovatoryctocara* are closely related to *Ovatoryctocara sinensis* from China (Supplementary Fig. S11S,T). *Paraantagmus latus* occurs with the youngest redlichiids in South China^73^. *Ovatoryctocara* was originally reported from Nevada by Sundberg & McCollum^17^ and subsequent discoveries have verified the occurrence of the genus as well as the *Oryctocarella* in the *A. arrojosensis* Biozone (ref.^39^, Fig. 4). Both genera occur below the FAD of *O. indicus* in South China^15,20,22,23,25,49,74^. In addition, *Euarthricocephalus* (Supplementary Fig. S11K,L) occurs with *O. indicus* in both Nevada and South China. *Bathynotus* (Supplementary Fig. S11A,X) occurs with olenellids and redlichiids in both Nevada and South China.

### Traditional lower–middle Cambrian boundaries

Recognition of the traditional lower–middle Cambrian boundary in Laurentia and China has a long history. In Laurentia, Walcott^75^ assumed that *Olenellus* and related taxa were originally middle Cambrian (=Georgia or Olenellus Fauna), present above the occurrence of Paradoxides Fauna (St. John Series in Newfoundland and Braintree Member in eastern Massachusetts). In 1890, Walcott^76^ recognized that the Olenellus Fauna was lower Cambrian and occurred below the Paradoxides Fauna. However, the nature of the boundary between the lower and middle Cambrian was obscure. Burling^8^ recognized the extinction of the olenellids marked the “Lower-Middle” Cambrian boundary^9^. The abruptness of the extinction led Cambrian workers^53,77,78^ to recognize this extinction event as the Olenellid Biomere boundary. Later, Palmer^79^ used the boundary to define the base of the Laurentian Delamaran Stage, Lincolnian Series.

In China, the occurrence of *Redlichia* was used to define the lower Cambrian, although not initially. Walcott (ref.^80^, p. 253) suggested that *Redlichia* was a direct descent of *Olenellus* and was upper lower or middle Cambrian in age. Walcott mentioned the similarity of *Redlichia* to *Zacanthoides* and the latter occurs above the Olenellus Fauna in western Utah and Nevada, further implying a middle Cambrian age. Walcott (ref.^81^, p. 2) discussed that Dames^82^ compared the fauna containing *Dorypyge richthofeni*^82^ as probably coeval with that of the Quebec Group, based on similarity of species from Utah. Walcott (ref.^83^, p. 4) also placed the boundary between the lower and middle Cambrian at the base of the Man To (Manto) Formation based on the occurrence of *Redlichia* because the genus “*is more closely related to Olenellus than to the trilobites of the Middle Cambrian fauna*.” Walcott^81^ assigned *R. finalis* to the middle Cambrian, but later he^83^ assigned the occurrence of *Redlichia* in China to the lower Cambrian based on the occurrence of the overlying trilobite assemblages. Walcott (ref.^83^, p. 2) stated that “*another important discovery was the occurrence in the Middle Cambrian of China of a fauna comparable with that of the Middle Cambrian of Mount Stephen, British Columbia, and the southern extension of the same fauna in the Middle Cambrian of Idaho, Utah, and Nevada in the United States*.” These middle Cambrian assemblages contain taxa with large pygidia, of which Walcott (ref.^83^, p. 53) considered important. Eventually, *Redlichia* was used to define the lower Cambrian in China because of its similarity to olenellids in Laurentia and the middle Cambrian was defined based on trilobite assemblages above *Redlichia* that had large pygidia similar to the trilobites found above the *Olenellus* Biozone in Laurentia.

During the 1960s and 1970s many trilobite workers were involved in geological mapping projects in China, and the concept that the extinctions of the olenellid and redlichiid faunas were synchronous began with Lu *et al*.^7^. Lu and others^7^ developed the bio-environmental control hypothesis and applied it to the Cambrian biostratigraphic correlation across the major cratons. Due to the lack of olenellid faunas in China, they suggested that the extinctions of the olenellid and redlichiid faunas were approximately synchronous. Thus, the traditional lower–middle Cambrian boundary in China was defined at the LAD of redlichiid trilobites (see Lu *et al*.^7^; see also the stratigraphic columns in ref.^6^). Chang (ref.^84^, p. 148; also see ref.^85^, p. 418) in his correlation of the Chinese and North American faunas and biozones stated that “redlichiid and olenellid trilobites […] disappeared simultaneously toward the end of the Early Cambrian”. This concept was further verified by the recognition of a strong δ^13^C_carb_ excursion in Laurentia and later in China. Strong shifts in δ^13^C_carb_ of the Cambrian oceans have been interpreted to be associated with extinction events^28,86,87^, thus, the extinctions of the olenellids and redlichiids have been linked^27,28,42,88–90^.

A paradox with the synchronous extinction idea was initially pointed out by Sundberg & McCollum (ref.^17^, p. 951). If the FAD of *O. indicus* is synchronous, then the extinction of the olenellids of Laurentia was prior to the extinctions of redlichiids in Gondwana. Alternatively, if extinctions of both olenellids and redlichiids are synchronous, the FAD of *O. indicus* may not be synchronous (as implied in ref.^18^, Fig. 2). Although the negative carbon shift (N2) is clearly associated with extinctions of both olenellids and redlichiids in both Laurentia and South China, both LADs of olenellids and redlichiids and associated carbon excursions show regional variations. Thus, this study supports the hypothesis that the FAD of *O. indicus* is the best marker to correlate the Laurentian sections to the base of the Wuliuan Stage and Miaolingian Series as defined at the GSSP in South China^18^.

In summary, Montañez *et al*.^27^ developed a δ^13^C_carb_ curve for the uppermost lower Cambrian to lower upper Cambrian (lower Furongian) of Laurentia using multiple stratigraphic successions from the Great Basin (Nevada and eastern California) and the southern Canadian Rockies. Since then, subsequent studies^41,42,60^ have been improving and refining the Cambrian chemostratigraphy for international correlation. Our study presents the most updated data for chemostratigraphy across the traditional lower–middle Cambrian boundary interval. Important highlights of this study are summarized below.

1. Based on trilobite biostratigraphy, our study indicates that the Cambrian Series 2–Miaolingian boundary coincides with the traditional lower–middle Cambrian boundary in South China and is very close to the traditional one in Laurentia.
2. The refined δ^13^C_carb_ record of Laurentia presented here (Supplementary Fig. S7) is consistent with that of Montañez *et al*.^27^ despite substantial differences in temporal resolution of the data sets and the older biostratigraphy derived from Palmer & Halley^40^ used by Montañez *et al*.^27^. The new data derived from the Miaobanpo section, in combination with previously published data, also support the presence of a negative carbon isotope excursion associated with the redlichiid extinction in South China (Supplementary Fig. S10).
3. In contrast to interpretations^18^ based on the GSSP section in South China indicating that both the gradual extinction of redlichiids and FAD of *O. indicus* (the current base datum for Wuliuan Stage) are linked together and they represent a single event, this study shows that the upper Olenellid Biomere boundary and FAD of *O. indicus* in Laurentia are two events separated by two trilobite biozones (Fig. 8), and the FAD of *O. indicus* is the best horizon to achieve correlation between the two regions.
4. The absence of a negative δ^13^C_carb_ excursion (N3 in Fig. 8) below the FAD of *O. indicus* in South China may imply stratigraphic condensation, a hiatus, and/or environmental changes in the South China sections that warrant further investigation.

## Materials and Methods

### Rock samples

Samples for carbonate (δ^13^C_carb_) and organic (δ^13^C_org_) carbon isotope analysis were collected over a series of years by the co-authors and analyzed at several laboratories. Samples from Nevada were collected from the *N. multinodus* to *P. denticulata* biozones in a platform-to-basin transect (Figs 1–3). This transect includes the outer shelf deposits at Split Mountain, middle shelf deposits at the Groom Range, and the inner shelf deposits at the Hidden Valley, Oak Spring Summit (δ^13^C_carb_ data from Faggetter *et al*.^42^), and Lyndon Gulch. These samples were originally collected to determine changes across the traditional lower–middle Cambrian boundary (the mutual *Nephrolenellus multinodus* Biozone and *Eokochaspis nodosa* Biozone boundary; Laurentian Dyeran Stage, Waucoban Series and Delamaran Stage, Lincolnian Series boundary).

Samples for δ^13^C_carb_ from South China were collected from the *Bathynotus kueichouensis-Ovatoryctocara sinensis* to *Peronopsis taijiangensis* biozones mainly from the Kaili Formation (Fig. 2). In China, three sections were studied: 1) Wuliu-Zengjiayan (ref.^44^; additional new samples), which is the GSSP of the Wuliuan Stage and Miaolingian Series; 2) Jianshan (ref.^44^); and 3) Miaobanpo. For the Miaobanpo section, samples cover only the five-meter interval below the FAD of *O. indicus*. Samples for δ^13^C_org_ were collected from the five-meter interval below the FAD of *O. indicus* at the Miaobanpo section and from the Kaili Formation at the Wuliu-Zengjiayan section. In an attempt to verify the results of Guo *et al*.^44^, we analyzed additional samples from the Wuliu-Zengjiayan section, specifically from the upper and lower portion of the Kaili Formation, base of the Jialao Formation, uppermost Tsinghsutung Formation and the interval around the FAD of *O. indicus* in 2010 to 2013. In addition, samples from the Miaobanpo section were analyzed to determine whether the strong negative δ^13^C_carb_ excursion near the FAD of *O. indicus* seen at the Jianshan section (ref.^44^) is present near the Wuliu-Zengjiayan section. Both δ^13^C_carb_ and δ^18^O_carb_ from all studied sections are plotted (Supplementary Fig. S1). Analytical procedures for carbonate stable isotope and organic carbon isotope analyses are included in the Electronic Supplementary Material.

### Compilation of chemostratigraphic profiles

The composite δ^13^C_carb_ and δ^13^C_org_ profiles were generated by recalculating the relative position of each data point in each biozone, which was rescaled based on the relative thicknesses and stratigraphic level (Figs 4–7; Supplementary Figs S2–S10). A LOESS analysis of the data using a 0.1 smoothing was used to generate the δ^13^C_carb_ and δ^13^C_org_ curves with 95% confidence bands for each section and consensus curve (ref.^91^). One problem encountered in using LOESS is that, if the section contains too few data points that are spread out stratigraphically, the 95% confidence levels cannot be accurately determined. In some cases (e.g., the δ^13^C_carb_ of the Groom Range section), increasing the smoothing factor to 0.2 provides some confidence intervals. However, in other cases (e.g., the δ^13^C_org_ of the Miaobanpo section) increasing the smoothing factor does not generate confidence levels. Therefore, those sections are illustrated only with a line connecting the data points (Supplementary Figs S9A and S10).

The Nevada stratigraphic successions vary in thickness reflecting differences in depositional environment. A multi-step method was used to construct the δ^13^C_carb_ and δ^13^C_org_ consensus curves. First, the data were divided into biozones to establish their relative position in the biozone. Second, each biozone was given a standard thickness in arbitrary units based on their relative thickness in individual sections. For example, the *N. multinodus* biozone was assigned to have 25 units and the overlying *E. nodosa* biozone was assigned to have 20 units. Third, the stratigraphic level of the carbon isotope data from each biozone for each section was recalculated based on the unit thickness of the biozone. These data were then stacked, so the top of the *N. multinodus* Biozone was at 25 units and the top of the *E. nodosa* Biozone was at 45 units. Fourth, the unit level of excursion events was adjusted according to incomplete sampling of a biozone (*N. multinodus* Biozone) and the presence of an unconformity within a biozone (e.g., *A. arrojosensis* Biozone, base of Susan Duster Limestone). On the other hand, the construction of the δ^13^C_org_ consensus curve for Nevada differs only in the last step where no distinctive pattern of shifts could be identified. The adjustment of stratigraphic positions from aligning excursions in the δ^13^C_carb_ curves were applied to the δ^13^C_org_ data.

The construction of the δ^13^C_carb_ summary curve for China differs only in the limited number of horizons that can be correlated in the sections studied. Key horizons used for correlation include the base of the Kaili Formation, *O. indicus* Biozone, and Jialao Formation. Only the lower half of the Kaili Formation was sampled in the Jianshan section. A composite δ^13^C_carb_ curve was generated for the three sections in this study (Fig. 7). The stratigraphic level of each data point was recalculated based on the biozone thicknesses at the Wuliu-Zengjiayan section. In addition, excursions were realigned to more closely match each other in the *Bathynotus kueichouensis-Ovatoryctocara sinensis* Biozone and the FAD of *O. indicus*.

## Supplementary information

Electronic Supplementary Material
