# Supplementary material for: Chemostratigraphic correlations across the first major trilobite extinction and faunal turnovers between Laurentia and South China
Source: Sci Rep. 2019 Nov 22;9:17392. doi: 10.1038/s41598-019-53685-2 (PMC6874646; doi:10.1038/s41598-019-53685-2)
Supplement: Supplementary file 1 — Electronic Supplementary Material [file 41598_2019_53685_MOESM1_ESM.pdf]

Supporting information for

Chemostratigraphic correlations across the first major trilobite extinction and faunal turnovers between Laurentia and South China

Jih-Pai Lin[1*], Frederick A. Sundberg[2], Ganqing Jiang[3], Isabel P. Montañez[4] and Thomas Wotte[5]

[1]*Department of Geosciences, National Taiwan University, P.O. Box 097, Taipei 10699, Taiwan*

[2]*Museum of Northern Arizona, 3101 N. Fort Valley Road, Flagstaff, AZ 86001, USA*

[3]*Department of Geoscience, University of Nevada, Las Vegas, NV 89154-4010, USA*

[4]*Department of Earth and Planetary Sciences, University of California, Davis, One Shields Avenue, Davis, CA 95616, USA*

[5]*Institut für Geologie, Technische Universität Bergakademie Freiberg, Bernhard-von-Cotta-Straße 2, D-09599 Freiberg, Germany*

Electronic Supplementary Material

***Corresponding author.**
Jih-Pai Lin (alexjplin@ntu.edu.tw; xyloplax1@gmail.com)

Contents of this file

Methods for isotope analyses
Chemostratigraphy of four sections in Nevada
Restudy of Oak Springs Summit section
Absolute age estimate
Oryctocephalus americanus versus *O. indicus*
Figures S1 to S11

Analytical methods for carbon and oxygen isotopes Carbonate carbon and oxygen isotope analysis

All samples for isotope analyses were cleaned and weathered crusts and calcite veins were avoided. Samples analyzed at the University of California, Davis (UCD) were micromilled and were roasted at 375°C under vacuum for 30 minutes to remove organics and subsequently reacted in 105% phosphoric acid at 90°C in either a common acid bath on a GVI Optima Stable Isotope Ration Mass Spectrometer or a Gilson Multicarbon Autosampler system interfaced with an Elementary Isoprime Mass Spectrometer. CO_2 gas was analyzed in dual inlet mode and the resulting values were corrected using an internal standard. Samples obtained at the University of Münster, Germany were pulverized using a tungsten carbide ring and puck mill. Material was analyzed for $\delta^{13}C_{carb}$ and $\delta^{18}O_{carb}$ using a Gasbench II connected via a ConFlow-III Interface to a Finnigan MAT DeltaPlusXL. Samples analyzed at the Las Vegas Isotope Science Laboratory of the University of Nevada, Las Vegas (UNLV) were microdrilled and reacted with orthophosphoric acid for 10 minutes at 70°C in a Kiel-IV Device automatically connected to a Finnigan DeltaPlus dual-inlet mass spectrometer. Precision monitored by NBS-19 calcite and an internal standard (UNLV, UCD & Münster) was < 0.1‰ for both $\delta^{13}C_{carb}$ and $\delta^{18}O_{carb}$ with the exception of $\delta^{18}O_{carb}$ (≤ 0.2‰) at the University of Münster lab. All delta values are reported relative to the VPDB standard.

Additional samples from the Wuliu-Zengjiayan section and the Miaobanpo section were collected using both a Dremel drill and/or a dental drill from clean, cut surface of each sample. Approximately 200 μg of sample powders were weighted out for carbonate carbon ($\delta^{13}C_{carb}$) and oxygen ($\delta^{18}O_{carb}$) analyses. Isotopes were analyzed with a Finnigan DeltaPlusXP system at the Earth Systems Center for Stable Isotopic Studies of Yale University (ESCSIS) and a Finnigan MAT252 at the Nanjing Institute of Geology and Palaeontology (NIGP). Reproducibility monitored by NBS-19 is better than 0.1‰ for both $\delta^{13}C_{carb}$ and $\delta^{18}O_{carb}$. Samples from the Miaobanpo section were also analyzed for organic carbon (kerogen) isotopes. Approximately 30 mg powders of each sample were collected and sent to a commercial lab for analyses.

Organic carbon isotope analysis

Carbon isotope analyses of organic matter ($\delta^{13}C_{org}$) occluded within the bulk rock (shales to shaley limestones) were carried out at two facilities at the University of California, Davis. Prior to powdering the

bulk rock samples by tungsten ball mill, the billets were trimmed to remove any observable weathering surfaces or fracture/secondary void filling calcites. In order to assess whether contamination from modern or extra-formational (i.e. hydrocarbons) organic matter has affected the measured $\delta^{13}C_{org}$ values, 40 shale samples were separated into two aliquots. One aliquot was treated solely with trace-element-grade hydrochloric acid (HCl), whereas the second aliquot was pretreated with dimethylchloride (DMC) following decarbonation using hydrochloric acid. Pretreating sedimentary rock samples characterized by low level TOC (total organic carbon) contents has been shown to be effective to remove labile, post-depositional organic carbon from kerogen. The majority of $\delta^{13}C_{org}$ values (72%) exhibit minimal difference (of ± 1‰ around zero) between samples extracted using HCl and samples prewashed with DMC prior to extraction with HCl indicating minimal effect of the DMC treatment. The difference between the two methods ($\Delta^{13}C_{org}$) can be expressed in the following equation (1):

$$\Delta^{13}C_{org} = \delta^{13}C_{org\text{-}HCl} - \delta^{13}C_{org\text{-}DMC} \qquad (1)$$

The remaining samples (28%) of the $\Delta^{13}C_{org}$ values range between 0.07 and 4.84‰ but do not show a consistent trend toward negative or positive $\Delta^{13}C_{org}$ values. Based on this experiment, subsequent samples were not pretreated with DMC and solely decarbonated using HCl. Sample Powders (~100 g) were repeatedly reacted with 10% trace-element-grade HCl until no reaction was observed (typically repeat the same procedure 2 to 4 times). Insoluble residues remaining after decarbonation were washed with deionized water (3 to 6 times) until solution pH was neutral, filtered, dried, and wrapped in tin capsules and analyzed using either 1) Isoprime continuous flow gas ratio mass spectrometer interfaced with an automated Carlo Erba elemental analyzer in the UC Davis Stable Isotope Laboratory or 2) a Europa Hydra 20/20 continuous flow IRMS at the Stable Isotope Facility in the Plant Sciences Department at UC Davis. Analysis of a subfraction of samples as replicates in both laboratories indicates no significant (≤ analytical uncertainty) interlaboratory variability. Long-term reproducibility for $\delta^{13}C_{org}$ is ≤ 0.3‰ based on repeated analysis of multiple internal standards and replicates.

Chemostratigraphy of four Cambrian sections in Nevada, USA

In the Groom Range section (Supplementary Fig. S3), $\delta^{13}C_{carb}$ values of the Combined Metals Member increase upward from −2.7‰ to −1.0‰ and then decrease to −4.2‰, with one data point of −5.0‰ just below the top of the *N. multinodus* Biozone. The $\delta^{13}C_{carb}$ values in the Susan Duster Limestone show an increase to +0.1‰, flanked by values of −1.2‰ in the uppermost *A. arrojosensis* and the basal *P. denticulata* biozones (uppermost Susan Duster Limestone). Most $\delta^{18}O_{carb}$ values fall between −14.5‰ and −12.0‰ and do not show covariation with $\delta^{13}C_{carb}$ (Supplementary Figs S1B, S3). In the Hidden Valley section (Supplementary Fig. S4), $\delta^{13}C_{carb}$ values of the Combined Metals Member decrease upward from −0.2‰ to −5.3‰ approximately five meters below the *N. multinodus*/*E. nodosa* boundary and then increase to about −1.0‰ in the uppermost layers of the member. Values in the Comet Shale Member show a slight decrease to −3.2‰ at the base with some values of approximately −1.5‰ immediately above. About one third through the member $\delta^{13}C_{carb}$ values vary between −3.9‰ and−1.5‰. From the top of the Comet Shale to the Susan Duster Limestone, within the upper *A. arrojosensis* Biozone, values increase from −4.3‰ to +0.3‰. In the basal *P. denticulata* Biozone, these higher values maintain but then decrease to −1.4‰ in the middle portion of the zone. In the Grassy Springs Member, within the lower *M. mexicana* Biozone, $\delta^{13}C_{carb}$ values increased to +0.6‰.

Most $\delta^{18}O_{carb}$ values of the upper Mule Spring Limestone to lowermost Emigrant Formation vary between −18.2‰ and −13.8‰ and maintain between −13.2‰ and –13.8‰ through the top of the *A. arrojosensis* Biozone. From the *P. praecurrens*/*G.walcotti* Biozones to the lower *Ehmaniella* Biozone, $\delta^{18}O_{carb}$ values begin at −13.2‰, decrease towards −16.2‰ in the lower portion, and then increase to −11.5‰ near the top of the section. The Hidden Valley section shows a relatively large range of $\delta^{18}O_{carb}$ values, varying from −24.7‰ to −8.7‰. The lower samples in the Combined Metals Member center between −16.0‰ and −15.0‰ and increase upward to −8.7‰ approximately five meters below the *N. multinodus*/*E. nodosa* boundary and then decrease to −15.4‰ in the uppermost layers of the member. Interestingly, the most negative $\delta^{13}C_{carb}$ values with relatively higher $\delta^{18}O_{carb}$ values from this interval were obtained from the thin calcite shells of trilobite fossils (yellow points in Supplementary Fig. S4). Approximately one third through the Comet Shale, $\delta^{18}O_{carb}$ values vary between −15.3‰ and −10.8‰. From the top of the Comet Shale to the Susan Duster Limestone, within the upper *A. arrojosensis* Biozone, $\delta^{18}O_{carb}$ values increase from −20.9‰ to −10.4‰. In the basal *P. denticulata* Biozone, these higher values

maintain roughly between −14.0‰ and −12.4‰ but then decrease to −16.8‰ in the lower portion of the zone and then increase to −13.9‰ in the middle portion of the member. In the Grassy Springs Member, within the lower *M. mexicana* Biozone, $\delta^{18}O_{carb}$ values increased to between −16.1‰ to −12.6‰.

In the Oak Spring Summit section (Supplementary Fig. S5) (data replotted from Faggetter *et al.*[1]), $\delta^{13}C_{carb}$ values of the Combined Metals Member increase upward from −4.0‰ to −1.0‰, then decrease to around −3.5‰ approximately five meters below the *N. multinodus*/*E. nodosa* boundary, and subsequently increase to −2.5‰ in the uppermost layers of the member. Values in the Susan Duster Limestone, within the upper *A. arrojosensis* Biozone and lowermost *P. denticulata* Biozone, increase to approximately 0.0‰. The Oak Spring Summit section shows $\delta^{18}O_{carb}$ values ranging from −18.0‰ to −8.5‰. The lower samples in the Combined Metals Member center around −18.0‰, increase upward to about −8.7‰ near the *N. multinodus*/*E. nodosa* boundary, and then decrease to around −15.0‰ at the Comet Shane/Susan Duster Limestone transition, within the upper *A. arrojosensis* Biozone and lowermost *P. denticulata* Biozone. Only a weak correlation ($r^2 = 0.24$) exists between $\delta^{13}C_{carb}$ and $\delta^{18}O_{carb}$ (Supplementary Fig. S1D).

In the Lyndon Gulch section (Supplementary Fig. S6), $\delta^{13}C_{carb}$ values of the Combined Metals Member increase upward from −3.8‰ to +0.1‰. Within the seven-meter interval below the *N. multinodus*/*E. nodosa* boundary, $\delta^{13}C_{carb}$ values decrease from 0.1‰ to minimum values of −3.4‰ to –3.7‰, and then increase to −0.4‰ in the lowermost Comet Shale. At the top of the Comet Shale and in the Susan Duster Limestone, within the upper *A. arrojosensis* Biozone, $\delta^{13}C_{carb}$ values increase from −2.0‰ to +0.7‰. In the middle portion of the Log Cabin Member, within the *P. denticulata* Biozone, $\delta^{13}C_{carb}$ values vary from −5.3‰ to −0.3‰, but overall they show a negative shift. In the Grassy Springs Member, within the lower *M. mexicana* Biozone, $\delta^{13}C_{carb}$ values increased to −0.1‰. The Lyndon Gulch section has $\delta^{18}O_{carb}$ values from −18.7‰ to −12.9‰, but there is no correlation between $\delta^{13}C_{carb}$ and $\delta^{18}O_{carb}$ (Supplementary Fig. S1E).

Restudy of Oak Springs Summit section

Of particular note is the section for Oak Springs Summit presented by Faggetter *et al.*[1] differs from the same section measured by Webster[2] and Linda & Mike McCollum (personal communication; Supplementary Fig. S5). Whereas the latter two measured sections agree, some significant differences between these measured sections and Faggetter *et al.*[1] include (Faggetter *et al.* versus Webster & McCollum): 1) shale and micrite

nodules below the oncolitic limestone in the Combined Metals Limestone versus sandstone; 2) nearly five meters of shale and micrite nodules above the oncolitic limestone versus two meters of covered interval (could be shale and micrite nodules); 3) nearly 15 m of shale and micrite nodules between the nodular limestone bed and a ribbon limestone (boundary limestone) versus 7 m of shale and micrite nodules; and 4) 20 m of shale versus nearly 30 m of shale between the boundary limestone and the Susan Duster Limestone. These inconsistencies do not invalidate the work of Faggetter *et al.*[1], but prevent an accurate correlation between the $\delta^{13}C_{carb}$ values generated by Faggetter *et al.*[1]and the $\delta^{13}C_{org}$ values determined in this study. In our sampled section (Supplementary Fig. S5), $\delta^{13}C_{org}$ values generally vary between −27.0‰ and −21.8‰ throughout the section. The Combined Metals Member illustrates a decrease in $\delta^{13}C_{org}$ from −21.8‰ to −25.5‰ below the upper contact and the *N. multinodus*/*E. nodosa* boundary. Above this interval, the $\delta^{13}C_{org}$ remains relatively constant between −25.2‰ and −22.8‰ and then varies between −25.3‰ and −27.0‰ in the upper Comet Shale. Both $\delta^{13}C_{carb}$ and $\delta^{13}C_{org}$ in the lower portion of the section show a general decrease in values. In contrast, $\delta^{13}C_{carb}$ and $\delta^{13}C_{org}$ seem to show opposite trends above the *N. multinodus* Biozone. However, given the lack of $\delta^{13}C_{carb}$ data in this portion of the section, such differences may be due to the limited $\delta^{13}C_{carb}$ data.

Absolute age estimate

According to Zhao *et al.*[3], the $^{206}Pb/^{238}U$ age estimate of 509.1±0.62 Ma for the base of Miaolingian Series and Wuliuan Stage is based on the refined dating of ash beds from the Comley Sandstone of United Kingdom (Ref.[4]). This date is older than the one we used for Laurentia (Ref.[5]) (Fig. 2) based on zircons recovered from Cambrian sections in the Grand Canyon. As a result, the maximum date for the extinction of olenellids and the traditional "lower−middle" Cambrian boundary of Laurentia is younger than 508 Ma. How much younger cannot be accurately ascertained. The next radiometric date above the 508 Ma are approximately 503 Ma from the Drumian Stage in Germany (Ref.[6]), Tasmania (Ref.[7]), and the United Kingdom (Ref.[4]).

Oryctocephalus americanus* versus *O. indicus

Zhao *et al.*[3,8,9] and Esteve *et al.*[10] suggested that *Oryctocephalus americanus* Sundberg & McCollum, 2003 (Ref.[11]) may be synonymous to *O. indicus* (Reed, 1910) (Ref.[12]), but they cannot be synonymized based on

the following reasons. *O. americanus* (Fig. S11H-J) and *O. indicus* (Fig. S11E-G) have similar cranidia, but the former differs from *O. indicus* in the number of transglabellar furrows (1 versus 3), broad-based, curved and flatten pleural spines (versus narrow-based, straight and rounded pleural spines), and fewer pygidial segments (2 versus 3). Furthermore, Zhao *et al.* (Ref.[8]; Ref.[3], p. 171) commented that the lack of two other transglabellar furrows in *O. americanus* is a result of a taphonomic bias, but we disagree (see Ref.[13]).

The second problem in the recent studies of *O. indicus* is the potential inclusion of other taxa in their studied specimens. Among trilobites that are co-occurring with *O. indicus* from the Kaili Formation, a sister taxon *O. elongatus* (Zhao, Ahlberg & Zhou, 1997) (Ref.[14]) is present, and this species includes *O. yui* Zhao & Yuan, in Yuan et al., 2002 (Ref.[15]) and possibly *O. sinicus* Zhao & Yuan, in Yuan et al., 2002 (Ref.[15]) (see Ref.[16]). *O. indicus* and *O. elongatus* also have similar cranidia, but the latter has only one strong T1 furrow and a broad-based, flattened pleural spines on the thorax and pygidium. One illustrated craidium in Esteve *et al.* (Ref.[10] , fig. 7g) is not *O. indicus* and probably belongs to *Curvoryctocephalus taijiangensis* Zhao & Yuan in Yuan et al., 2002 (Ref.[15]).

The third issue is the taphonomic effect on glabellar furrows. Esteve *et al.*[17] explored the development of the transglabellar furrows in *O. indicus* based on specimens from Siberia (=*O. renticulatus*), South China, and North America, but *O. americanus* was not compared or discussed in their study. While they proposed that the difference in the depth and shape of the transglabellar furrows is the result of preservation, all three samples in general have three transglabellar furrows. A recent study on another related taxon *Oryctocephalites palmeri* Sundberg & McCollum, 1997 (Ref.[18]) illustrates that compaction enhances furrow depth between glabellar pits (Ref.[13]). Therefore, transglabellar furrow is the most critical feature to distinguish *O. indicus* from other sister taxa. Illustration of cranidia with poor preservation of transglabellar furrow (e.g., Ref.[3], fig. 6a) is not useful for precise identification; thus, is not recommended.

The fourth issue is the diachronous nature of key trilobite ranges in different cratons. Although *O. americanus* from Laurentia is similar to *O. elongatus* from South China by bearing one transglabellar furrow (T1), the former ranges below the FAD of *O. indicus* in the *Amecephalus arrojosensis* Biozone and *O. indicus* Biozone (Fig. 8) and the latter occurs in within the *O. indicus* Biozone[15,16].

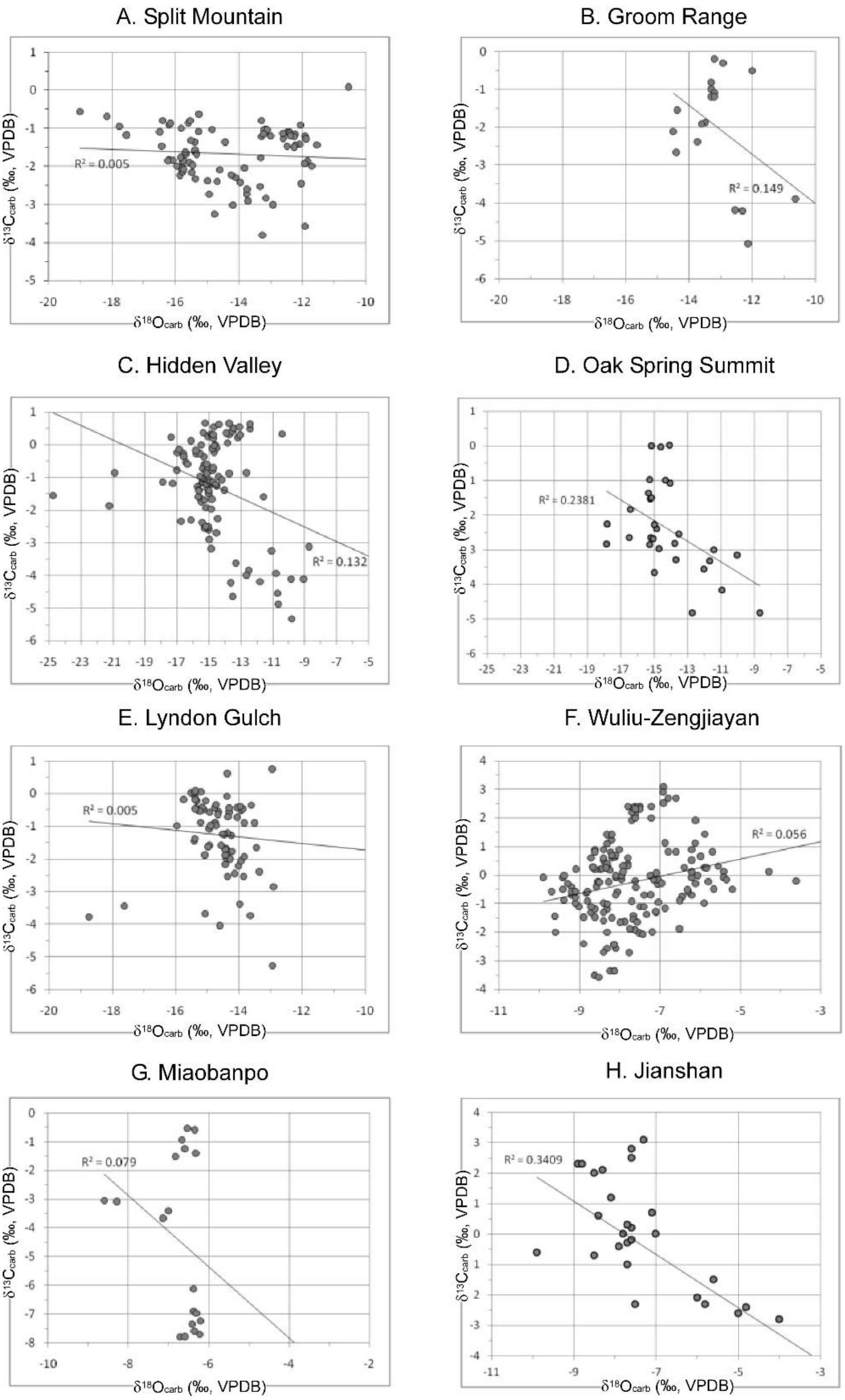

Fig. S1. The cross plots of $\delta^{18}O_{carb}$ versus $\delta^{13}C_{carb}$ data from all studied sections in both Nevada (A–E) and South China (F–H). A, Split Mountain section. B, Groom Range section. C, Hidden Valley section. D, Oak Spring Summit. E, Lyndon Gulch section. F, Wuliu-Zengjiayan section. G, Miaobanpo section. H, Jianshan section (see Fig. 1 for location map).

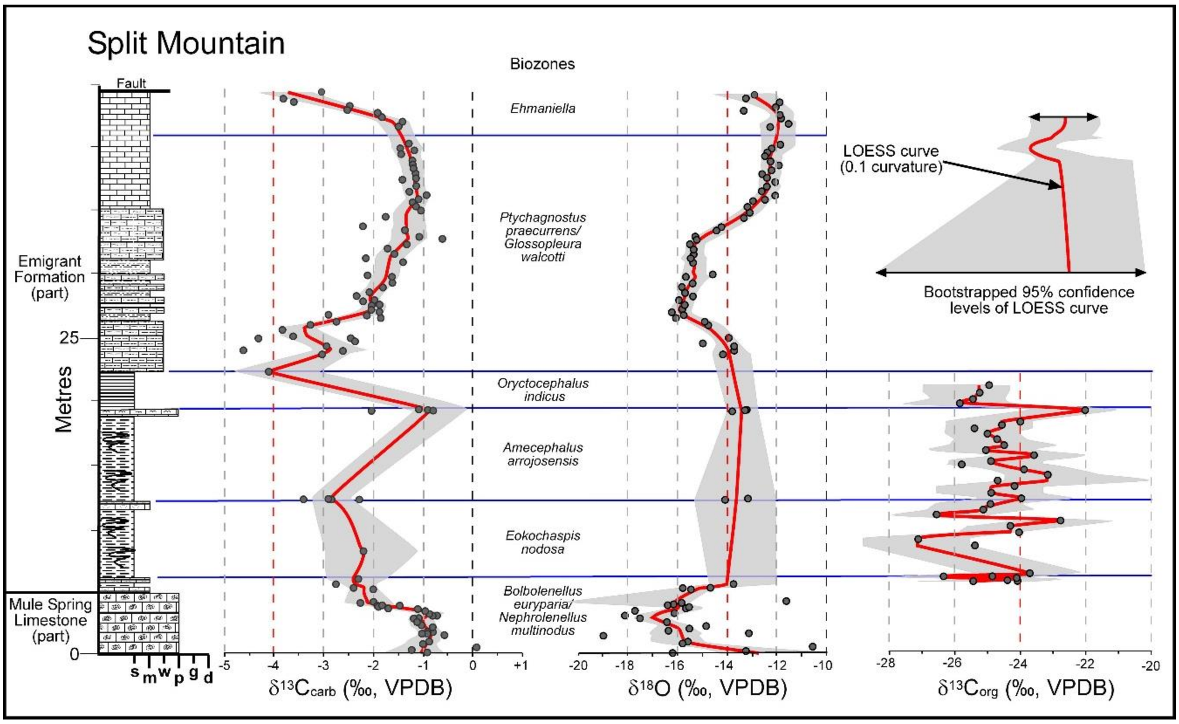

Fig. S2. Carbon and oxygen isotopic results from the Split Mountain section, Nevada.

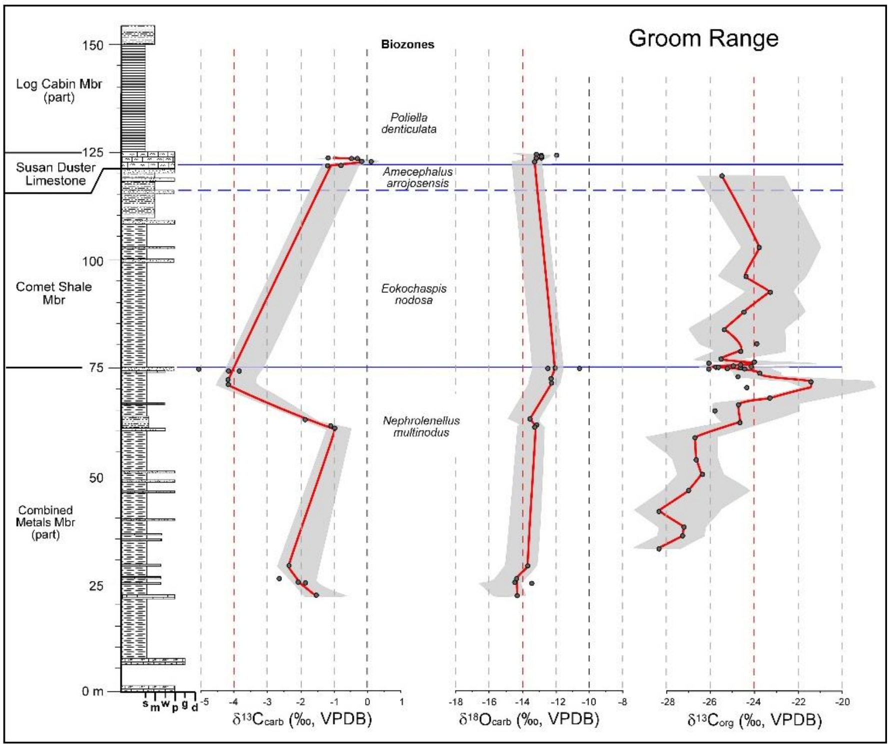

Fig. S3. Carbon and oxygen isotopic results from the Groom Range section, Nevada. LOESS curve and confidence levels are based on 0.2 curvature value.

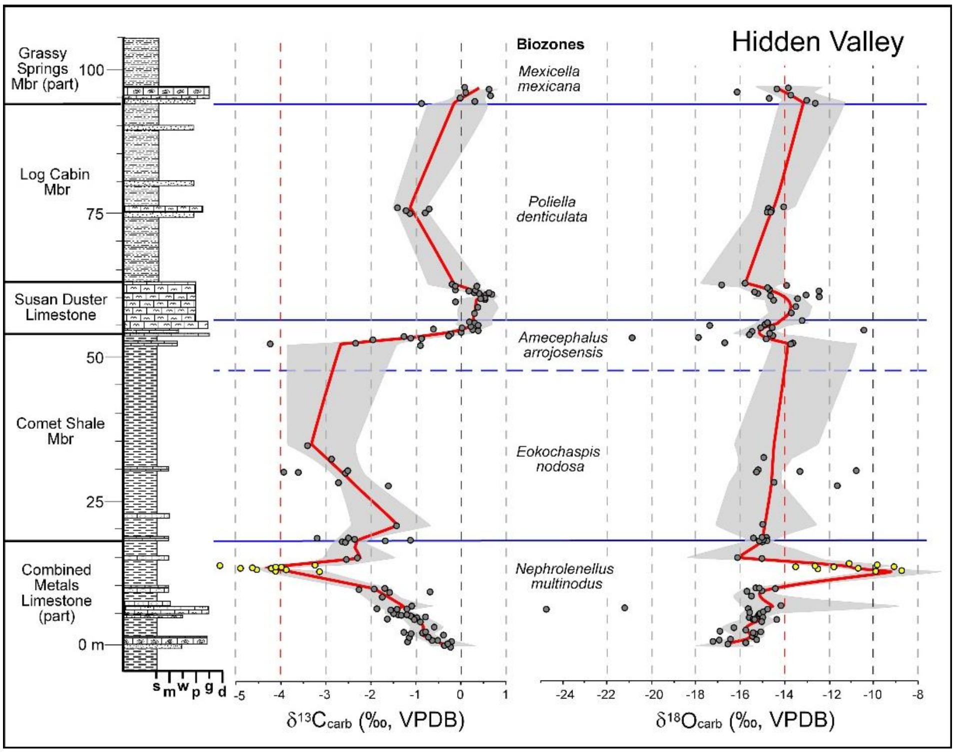

Fig. S4. Carbon and oxygen isotopic results from the Hidden Valley section, Nevada. Yellow points are values generated from trilobite exoskeletons.

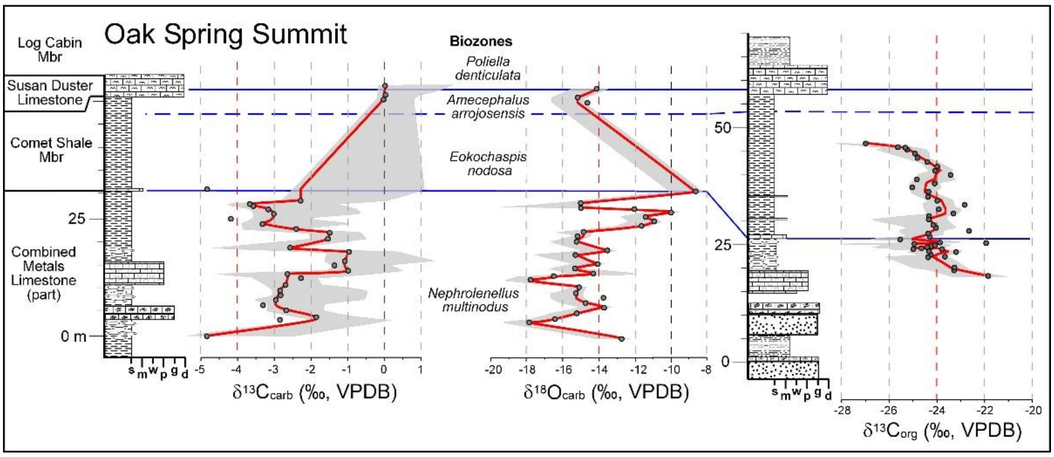

Fig. S5. Carbon and oxygen isotopic results from the Oak Spring Summit section, Nevada. The $\delta^{13}C_{carb}$ and $\delta^{18}O_{carb}$ data are plotted against the stratigraphic column redrawn from Faggetter *et al.*[1]; which is also the source of data. The $\delta^{13}C_{org}$ data is based on measurements from Webster[2] and L.B. & M. McCollum (personal communications; see text for discussion).

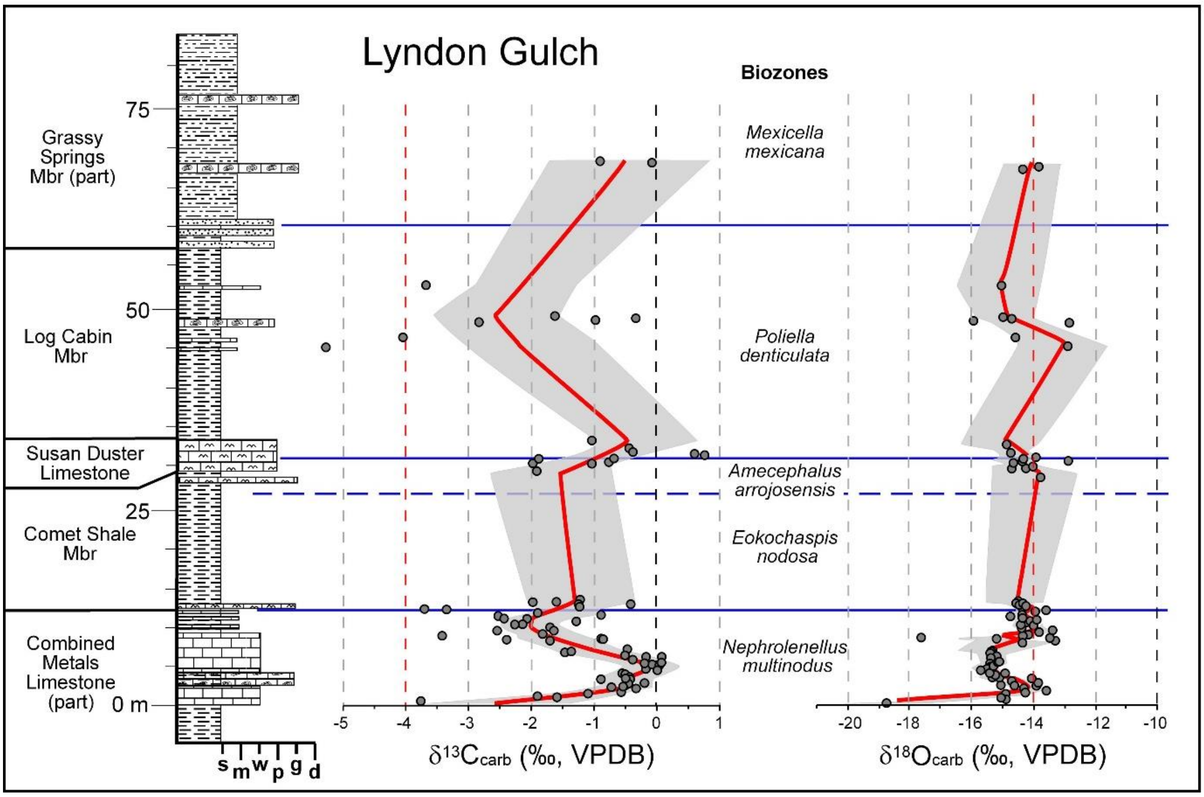

Fig. S6. Carbon and oxygen isotopic results from the Lyndon Gulch section, Nevada.

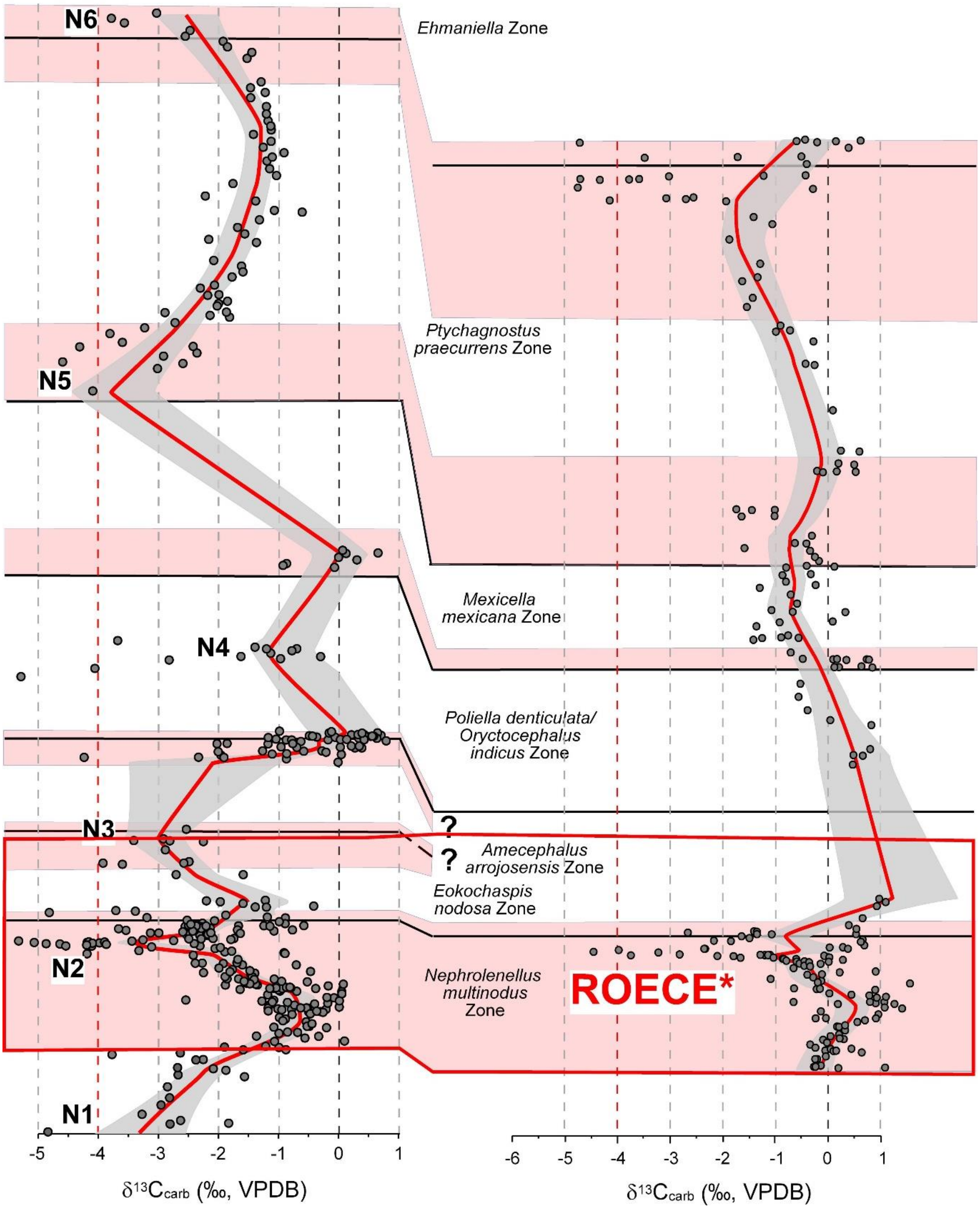

Fig. S7. Comparison of the $\delta^{13}C_{carb}$ summary curve to a portion of the Cambrian curve by Montañez *et al.* (Ref.[19] , fig. 4; data replotted).

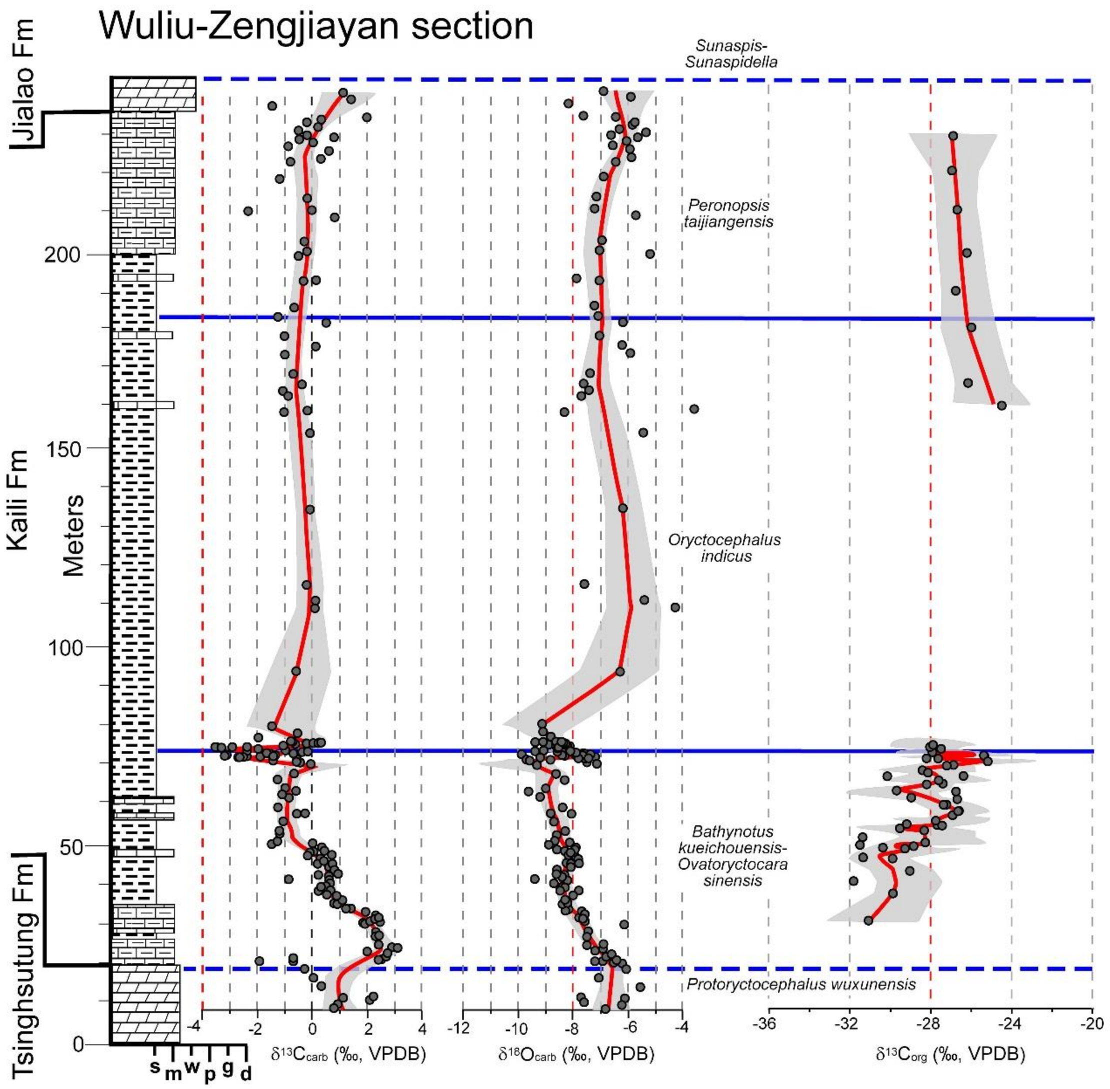

Fig. S8. Carbon and oxygen isotopic results from the Wuliu-Zengjiayan section, South China.

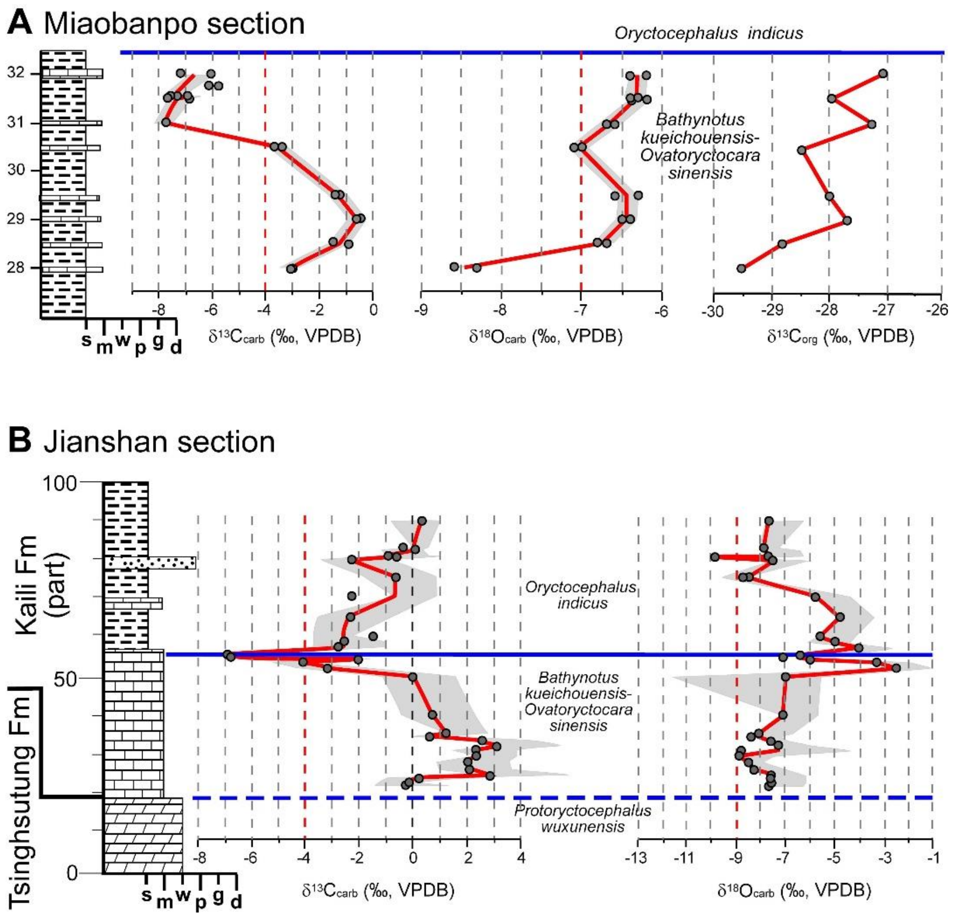

Fig. S9. Carbon and oxygen isotopic results from two sections in South China. A, Data from the barren zone between the LAD of *Bathynotus* sp. and the FAD of *Oryctocephalus indicus* at the Miaobanpo section, South China. B, Data from the Jianshan section, South China (replotted from Ref.[20]).

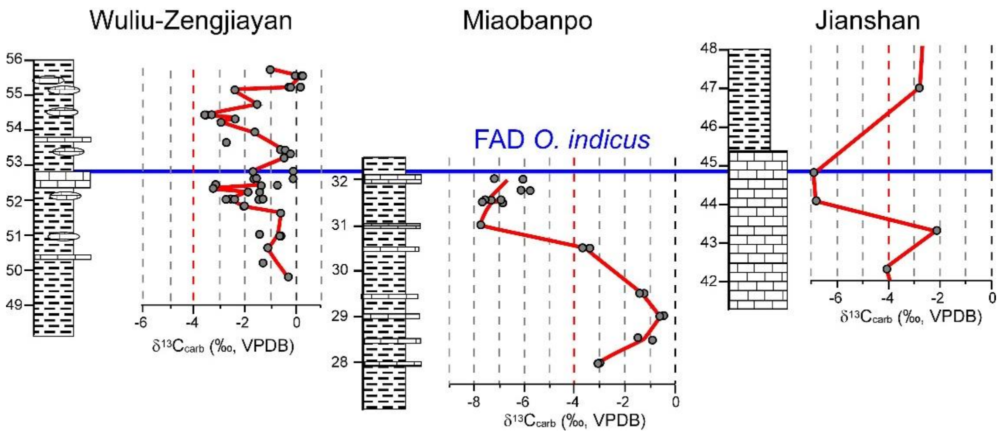

Fig. S10. Chemostratigraphic correlation of the Wuliuan Stage and Miaolingian Series boundary interval (indicated by the FAD of *O. indicus*) based on $\delta^{13}C_{carb}$ values for the three stratigraphic sections in South China.

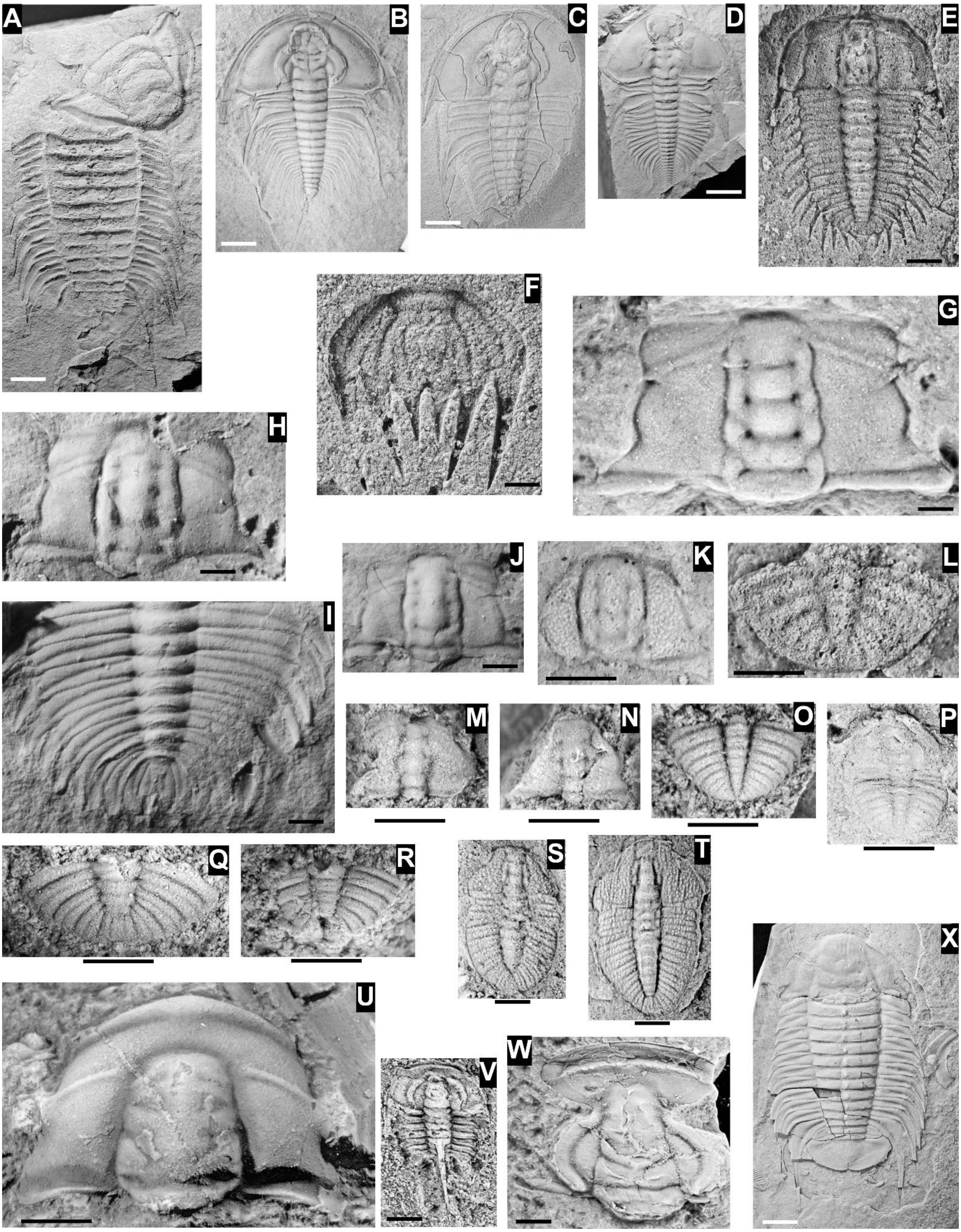

Fig. S11. Key trilobites from the lower Wuliuan Stage and Miaolingian Series boundary interval. All specimens are deposited in the United States National Museum of Natural History (USNM), Field Museum of Natural History, Chicago (FMNH), Institute for Cambrian Studies, University of Chicago (ICS), and Museum of College of Resource and Environment Engineering, Guizhou University (GK; localities FZX;

see Sundberg *et al.*[16]). White scale bars = 5 mm, Black scale bars = 1 mm. Figures A–D, X provided by Mark Webster; E, F provided by Jorge Esteve and the specimens are housed at the Museo de Ciencias Naturales de la Universidad de Zaragoza (Ref.[21]); A) *Bathynotus granulatus* Lermontova, 1940 (Ref.[21]), cranidium is an internal mold and thorax and pygidium are external molds (FMNH 58120), *Nephrolenellus multinodus* Biozone, Combined Metals Member, Pioche Shale, Nevada; B) *Olenellus gilberti* (Meek in White 1874) (Ref.[21]), internal mold of exoskeleton (FMNH 58120) *N. multinodus* Biozone, Combined Metals Member, Pioche Shale, Nevada; C) *Olenellus terminatus* Palmer 1998 (Ref.[21]), internal mold of exoskeleton (ICS 1044-2), *N. multinodus* Biozone, Combined Metals Member, Pioche Shale, Nevada; D) *Nephrolenellus geniculatus* Palmer 1998 (Ref.[21]), *N. multinodus* Biozone, Combined Metals Member, Pioche Shale, Nevada; E–G) *Oryctocephalus indicus* (Reed 1910) (Ref.[21]) from mudstones within 1 m above the FAD of *O. indicus*, Emigrant Formation, Nevada; E), internal mold of exoskeleton; MPZ 2019/38; F) internal mold of pygidium, MPZ 2019/387; G) cranidium, USNM726200; H-J) *Oryctocephalus americanus* Sundberg & McCollum, 2003 (Ref.[21]) from mudstones below the base of the *O. indicus* Biozone, Emigrant Formation, Nevada; H) cranidium, USNM 517577 (USNM loc. 41964); I) thorax and pygidium, USNM 517580 (USNM loc. 41976); J) cranidium, USNM 517587 (USNM loc. 41984); K, L) *Euarthricocephalus nevadensis* (Sundberg and McCollum 2003) (Ref.[21]) from mudstones ca. 25 cm above base of the *O. indicus* Biozone, Emigrant Formation, Nevada; K) latex cast of cranidium, USNM 488938 (USNM loc. 41078, Goldfield Hills); L) latex cast of pygidium, USNM 488947 (USNM loc. 41078, Goldfield Hills); M-P) *Ovatoryctocara* cf. *sinensis* Zhao et al. 2015 (Ref.[21]) from the bioclastic limestone bed directly below the base of the *O. indicus* Biozone, Emigrant Formation, Nevada; M) cranidium, USNM 726201 (USNM loc. 41985); N) cranidium, USNM 727602 (USNM loc. 41985); O) pygidium, USNM 726203 (USNM loc. 41985); P) small articulated shield in shale below the bioclastic limestone, USNM 726204 (USNM loc. 41984); Q, R) *Oryctocarella* sp. from the bioclastic limestone bed directly below the base of the *O. indicus* Biozone, Emigrant Formation, Nevada; Q) pygidium, USNM 726205 (USNM loc. 41985); R) partial pygidium, USNM 728213 (USNM loc. 41985); S, T) *Ovatoryctocara sinensis* Zhao et al. 2015 (Ref.[21]), *Bathynotus kueichouensis-Ovatoryctocara sinensis* Biozone, Kaili Formation, Guizhou, China; S) latex cast of external mold of shield (GK B3 0090; FZX34); T) latex cast of external mold of shield (GK B3 0091; FZX42); U) *Paraantagmus latus* Yuan & Li 1999 (Ref.[21]) from the limestone bed approximately 6 m below

the base of the *O. indicus* Biozone, Emigrant Formation, Nevada, cranidium, USNM 517596 (USNM loc. 41953); V, W) *Redlichia* (*Redlichia*) *takooensis longspina* Guo & Zhao 1998 (Ref.[21]), *Bathynotus kueichouensis-Ovatoryctocara sinensis* Biozone, Kaili Formation, Guizhou, China; V) partial shield, exfoliated (GK B3 0030; loc. FZX32); W) cranidium, partially exfoliated (GK B3 0028; loc. FZX39); X) *Bathynotus kueichouensis* Lu in Wang 1964 (Ref.[21]), mostly testate shield (FMNH PE58139a), *Bathynotus kueichouensis-Ovatoryctocara sinensis* Biozone, Kaili Formation, Guizhou, China.

References

1 Faggetter, L. E. *et al.* Trilobite extinctions, facies changes and the ROECE carbon isotope excursion at the Cambrian Series 2−3 boundary, Great Basin, western USA. *Palaeogeogr. Palaeoclimatol. Palaeoecol.* **478**, 53−66 (2017).

2 Webster, M. Trilobite biostratigraphy and sequence stratigraphy of the Upper Dyeran (traditional Laurentian "Lower Cambrian") in the southern Great Basin, U.S.A. *Mus. North. Ariz. Bull.* **67**, 121−154 (2011).

3 Zhao, Y. *et al.* Global Standard Stratotype-Section and Point (GSSP) for the conterminous base of the Miaolingian Series and Wuliuan Stage (Cambrian) at Balang, Jianhe, Guizhou, China. *Episodes* **42**, 165−84 (2019).

4 Harvey, T. H. P. *et al.* A refined chronology for the Cambrian succession of southern Britain. *J. Geol. Soc.* **168**, 705−716 (2011).

5 Karlstrom, K. *et al.* Cambrian Sauk transgression in the Grand Canyon region redefined by detrital zircons. *Nature Geoscience* **11**, 438−443 (2018).

6 Landing, E., Geyer, G., Buchwaldt, R. & Bowring, S. A. Geochronology of the Cambrian: a precise Middle Cambrian U-Pb zircon date from the German margin of West Gondwana. *Geol. Mag.* **152**, 28−40 (2015).

7 Perkins, C. & Walshe, J. L. Chronology of the Mount Read Volcanics, Tasmania, Australia. *Econ. Geol.* **88**, 1176−1197 (1993).

8 Zhao, Y.-L. *et al.* A new section of Kaili Formation (Cambrian) and a biostratigraphic study of the boundary interval across the undefined Cambrian Series 2 and Series 3 at Jianshan, Jianhe County, China with a discussion of global correlation based on the first appearance datum of *Oryctocephalus indicus* (Reed, 1910). *Prog. Nat. Sci.* **18**, 1549−1556 (2008).

9 Zhao, Y. L., Esteve, J., Yuan, J.-L., Peng, J. & Sun, H.-J. Taphonomy and morphologic variation of *Oryctocephalus indicus* from China, Russia and USA. in *The Second International Congress on Stratigraphy, Strati 2015* (eds E. Gülli & W. E. Piller) 430 (Ber. Ins. Erdwiss. K.-F.-Univ. Graz, 21, 2015).

10 Esteve, J., Zhao, Y.-L. & Peng, J. Morphological assessment of the Cambrian trilobites *Oryctocephalus indicus* (Reed 1910) from China and *Oryctocephalus 'reticulatus'* (Lermontova 1940) from Siberia. *Lethaia* **50**, 175−193 (2017).

11 Sundberg, F. A. & McCollum, L. B. Trilobites of the Lower Middle Cambrian *Poliella denticulata* Biozone (New) of southeastern Nevada. *J. Paleontol.* **77**, 331−359 (2003).

12 Reed, F. R. C. The Cambrian Fossils of Spiti. *Mem. Geol. Soc. India, Palaeontol. Indica, Series XV* **7**, 1−71 (1910).

13 Webster, M. & Sundberg, F. A. Nature and significance of intraspecific variation in the early Cambrian oryctocephalid trilobite *Oryctocephalites palmeri* Sundberg and McCollum, 1997. *J. Paleontol.* (In press).

14 Zhao, Y. L., Ahlberg, P. & Zhou, Z. The *Oryctocephalina* in Kaili Formation, Guizhou. *J. Guizhou Univ. Technol.* **26**, 32−36 (1997).

15 Yuan, J.-L., Zhao, Y.-L., Li, Y. & Huang, Y.-Z. *Trilobite Fauna of the Kaili Formation (uppermost Lower Cambrian−lower Middle Cambrian) from southeastern Guizhou, South China*. (Shanghai Science and Technology Press, 2002).

16 Sundberg, F. A., Zhao, Y.-L., Yuan, J.-L. & Lin, J.-P. Detailed trilobite biostratigraphy across the proposed GSSP for Stage 5 ("Middle Cambrian" boundary) at the Wuliu-Zengjiayan section, Guizhou, China. *Bull. Geosci.* **86**, 423−464 (2011).

17 Esteve, J., Zhao, Y. L., Maté-González, M. A., Gómez -Heras, M. & Peng, J. A new high-resolution 3-D quantitative method for analysing small morphological features: an example using a Cambrian trilobite. *Sci. Rep.* **8**, 2868 (2018).

18 Sundberg, F. A. & McCollum, L. B. Oryctocephalids (Corynexochida: Trilobita) of the Lower-Middle Cambrian Boundary Interval from California and Nevada. *J. Paleontol.* **71**, 1065−1090 (1997).

19 Montañez, I. P., Osleger, D. A., Banner, J. L., Mack, L. E. & Musgrove, M. Evolution of the Sr and C isotope composition of Cambrian oceans. *GSA Today* **10**, 1−7 (2000).

20 Guo, Q.-J. *et al.* A negative carbon isotope excursion defines the boundary from Cambrian Series 2 to Cambrian Series 3 on the Yangtze Platform, South China. *Palaeogeogr. Palaeoclimatol. Palaeoecol.* **285**, 143−151 (2010).

21 Canudo, J. I. The collection of type fossils of the Natural Science Museum of the University of Zaragoza (Spain). *Geoheritage* **10**, 385−392 (2018).
